# Somatic mutational landscape in von Hippel–Lindau familial hemangioblastoma

**DOI:** 10.1002/1878-0261.70228

**Published:** 2026-03-23

**Authors:** Maja Dembic, Anne Nørremølle, Lilian Bomme Ousager, Lars van Brakel Andersen, Marie Louise Mølgaard Binderup, Mads Thomassen

**Affiliations:** ^1^ Clinical Genome Center, Department of Clinical Research University of Southern Denmark Odense Denmark; ^2^ Department of Clinical Genetics Odense University Hospital Odense Denmark; ^3^ Department of Cellular and Molecular Medicine University of Copenhagen Copenhagen Denmark

**Keywords:** copy number, hemangioblastoma, single nucleotide, somatic, *VHL*, WES

## Abstract

Von Hippel–Lindau disease (vHL) predisposes to tumor development, mainly clear cell renal carcinoma and hemangioblastoma. The underlying cause is germline variants in the *VHL* gene, with tumorigenesis thought to require additional somatic ‘second‐hit’ events that most commonly include loss of 3p. However, the precise mechanisms of vHL‐related tumor development remain incompletely understood. Genomic investigations of familial hemangioblastoma may help elucidate the early steps of tumorigenesis and contribute to improved disease prediction, biomarker discovery, and therapeutic strategies. We performed whole exome sequencing on 22 familial hemangioblastomas from 7 patients representing 5 unrelated families, and with 4 different causative *VHL* genotypes. The tumors exhibited low overall mutational burden but showed frequent loss of heterozygosity on chromosome 3 or 3p and single nucleotide variants in the *VHL* region. Variants were significantly enriched in genes associated with GABAergic and serotonergic neuronal cell types, as well as in pathways regulating cell cycle and neurogenesis. These findings suggest that, in addition to *VHL* loss, dysregulation of neuronal differentiation programs and cell cycle control may play important roles in hemangioblastoma tumorigenesis.

## Graphical abstract

The causes of central nervous system (CNS) hemangioblastoma in Von Hippel–Lindau (vHL) disease are unclear. We used Whole Exome Sequencing (WES) on familial hemangioblastoma to investigate events that underlie tumor development. Our findings suggest that *VHL* loss creates a permissive environment for tumor formation, while additional alterations contribute to selective pressures on specific developmental and cell cycle‐related pathways in certain cell types, such as GABAergic and serotonergic neural cell types.

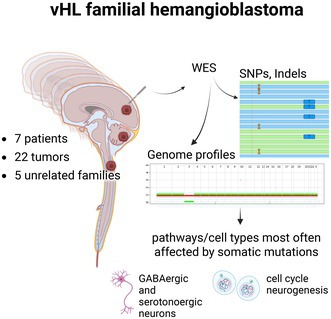

**Abbreviations**
ASCAT: Allele‐Specific Copy Number Analysis of Tumors
BAM: binary alignment map
*BAP1*
: *BRCA1* associated protein 1 gene
CADD: Combined Annotation Dependent Depletion
CaVEMan: Cancer Variants Through Expectation Maximization
ccRCC: clear cell renal carcinoma
CNS: central nervous system
CNV: copy number variant
dbNSFP: database for nonsynonymous SNPs' functional predictions
FDR: false discovery rate
FFPE: formalin‐fixed and paraffin embedded
GABA: gamma aminobutyric acid
GATK: The Genome Analysis Toolkit
GnomAD: The Genome Aggregation Database
GO: gene ontology
GOBP: gene ontology biological process
GOCC: gene ontology cellular component
GOMF: gene ontology molecular function
GSEA: gene set enrichment analysis
HGABA: human GABAergic neuron
HIF: hypoxia inducible factors
HNBGABA: human GABAergic neuroblast
HNBML5: mediolateral neuroblast group 5
HPROGFPL: human neuronal progenitor of the lateral floorplate.
HSERT: human serotonergic neuron
Indel: small insertion or deletion
LOH: loss of heterozygosity
MSigDB: The Molecular Signature Database
*NIN*
: Ninein gene
*PBRM1*
: carcinoma polybromo 1 gene
PHRED: Phil's Read Editor score
pVHL: VHL protein
*SETD2*
: SET domain containing 2 gene
SNP: single nucleotide polymorphism
SNV: single nucleotide variant
SOB: strand orientation bias
*TTN*
: Titin gene
VAF: variant allele frequency
vHL: Von Hippel–Lindau disease
*VHL*
: Von Hippel–Lindau gene
WES: whole exome sequencing

## 1 Introduction

Patients with the autosomal dominant hereditary disease von Hippel–Lindau (vHL) (MIM # 193300) are at risk of lifelong tumor development in multiple organs, mainly renal cell carcinomas and central nervous system (CNS) hemangioblastomas [1, 2]. CNS hemangioblastoma development, both sporadic and vHL‐related, is characterized by unpredictable periods of growth and stagnation [3, 4, 5]. vHL is most often caused by germline variants in the tumor suppressor *VHL* gene. In approximately 20% of vHL patients, the disease is sporadic; these patients usually have a *de novo VHL* variant and no relevant family history [2]. The diagnosis of vHL in patients with a positive family history can be based on only one tumor, while in sporadic cases it requires the presence of at least two tumors [2]. Sporadic cases represent a diagnostic challenge as *de novo* variants may result in disease mosaicism. Affected individuals may present with clinical features of the disease, yet test negatively on genetic screening, since the variant may not be present in all peripheral leucocytes [4].

The vHL disease is caused by a disrupted function of the VHL protein (pVHL), which plays a crucial role in the cellular oxygen‐sensing pathway, a discovery that in 2019 led to the award of the Nobel Prize in Physiology or Medicine to William G. Kaelin Jr., Sir Peter J. Ratcliffe, and Gregg L. Semenza [6]. pVHL is part of an ubiquitin ligase complex, which uses oxygen as a cosubstrate to mediate the proteasomal degradation of a specific group of transcription factors, called hypoxia inducible factors (HIFs). During hypoxia, or if pVHL is absent or inactive, HIFs are stabilized activating a response to hypoxia. HIFs induce a wide range of target genes implicated in processes such as angiogenesis, proliferation, apoptosis, and metabolism [7]. The VHL protein is also involved in HIF‐independent processes, potentially involved in tumor formation: pVHL regulates extracellular matrix composition and the formation of microtubules, it activates and stabilizes the p53 protein, and induces apoptosis in neuronal cells [7].

The vHL disease is associated with high morbidity and mortality and has an almost complete penetrance at 60–75 years [8, 9]. Although genotype–phenotype correlations have been established in vHL, the clinical course of the disease can be variable. Even among patients with the same pathogenic germline variant there are variations in tumor burden, age at which the tumors appear, as well as patterns of tumor growth. The relative unpredictability of the clinical course complicates patient management and causes a high psychological burden for affected families [2].

The mechanisms behind vHL‐associated tumorigenesis are complex and remain incompletely understood. In general, tumor formation occurs when a somatic variant inactivates the remaining wild‐type allele in susceptible target tissue, resulting in complete loss of a functional VHL protein [5]. The *VHL* gene maps to the short arm of chromosome 3 (3p25), and a second‐hit event preceding tumorigenesis will typically occur via different mechanisms, which may include methylation of the promoter, point or small mutations, loss of 3p or of the entire chromosome 3 [10]. Among these, the most common type of second hit has been reported to be loss of 3p [11, 12]. However, while inactivation of both copies of the *VHL* gene is regarded as necessary, it may not be sufficient on its own to drive hemangioblastoma development [1]. Bi‐allelic *VHL* inactivation may be present in multiple tumor precursor foci throughout the predisposed tissues, but most of them never develop into actual symptom‐causing tumors [13, 14, 15]. Therefore, to gain a deeper understanding of the mechanisms underlying vHL‐associated tumorigenesis, it is important to identify the additional factors that contribute to tumor initiation and progression. We hypothesized that CNS hemangioblastomas exhibit shared genetic alterations that contribute to tumorigenesis in pVHL‐deficient cells. To explore the mechanisms underlying tumor development in vHL‐associated familial hemangioblastomas, we analyzed the somatic mutational landscape of 22 surgically resected CNS hemangioblastomas from 7 patients with vHL disease.

## 2 Materials and methods

### 2.1 Patients

Through the national vHL Database, we identified vHL patients who had a reported pathogenic *VHL* germline variant, who had undergone surgical removal of at least two histopathologically confirmed CNS hemangioblastomas, thereby maximizing the likelihood of obtaining tissue from multiple tumors of the same individual. We initially identified 13 vHL patients with a total of 48 reported surgically removed CNS hemangioblastomas, who all gave their informed consent for the collection of clinical data and tumor‐related information (medical records including imaging and histopathological reports) as well as any tumor samples from pathology departments. The biological samples were collected from pathological departments over a period of 2010–2015 at the Institute for Cellular and Molecular Medicine, University of Copenhagen. The tumors were removed during surgeries from 1981 to 2013. We were able to obtain formalin‐fixed and paraffin embedded (FFPE) samples and clinical information from 32 hemangioblastomas from 8 patients. Blood EDTA samples were collected from these 8 patients. The paraffin‐embedded samples were sectioned to 10 μm of thickness with steel knives using a sledge microtome. Hematoxylin and Eosin (H&E) stained sections of each tumor sample were examined under light microscope and evaluated by a pathologist to confirm the pathological diagnosis of hemangioblastoma.

Genomic DNA was extracted from blood and FFPE samples using Maxwell® DNA FFPE isolation kit and Maxwell® Blood DNA kit (Promega, USA). After extraction, DNA concentration was checked on a Nanodrop. Four hemangioblastoma samples had to be excluded due to low DNA yield (less than 300 ng total DNA in 90 μL). After sequencing, three samples were excluded due to overall low sequence quality, and three additional samples were excluded as the genotype did not match the one obtained from the blood sample. In total, we analyzed the sequences of 22 tumor samples from 7 patients. Tumor‐related clinical data were evaluated for tumors whose samples could be successfully analyzed.

### 2.2 Ethics approval and consent to participate

The study was conducted in accordance with the principles of the Declaration of Helsinki. The protocol was approved by the Regional Ethics Committee (H‐19016881) and the Danish Data Protection Agency. All patients gave their written informed consent to be included in the study prior to sample collection. For enquiries about the possibility of using the biological material included in this study, please refer to M.L.M.B. Further research investigations on the biological material may require new ethics committee approvals.

### 2.3 Whole exome sequencing and data analysis

In order to identify somatic variants in the tumors, we performed whole exome sequencing (WES) on matched blood and FFPE samples. The libraries were prepared using Roche NimbleGen SeqCap EZ MedExome Enrichment Kits. 2 × 100 bp pair end sequencing was performed on an Illumina NextSeq 550 platform.

Raw reads were processed using BWA‐MEM v.0.7.12 and aligned to the reference sequence (human NCBI build 37). The Genome Analysis Toolkit (GATK v.4.2.3.0) was used to remove duplicates, recalibrate base quality scores, and generate recalibrated binary alignment map (BAM) files. For somatic variant calling of tumor‐blood matched samples, we used three different variant callers: Mutect2, CaVEMan, and Pindel to collect as much variation as possible. Mutect2 and CaVEMan (Cancer Variants Through Expectation Maximization: http://cancerit.github.io/CaVEMan/) were used for calling somatic and germline single nucleotide variants (SNVs). A lightly modified version of Pindel 2.0 (http://cancerit.github.io/cgpPindel/) was used for calling somatic and germline small insertions or deletions (indels). The specific versions of the tools used are found in the cgpwgs‐2.1.0 docker image (https://dockstore.org/containers/quay.io/wtsicgp/dockstore‐cgpwgs:2.1.0).

Raw data quality check was done using FastQC. After data processing, Qualimap and Picard (CollectMultipleMetrics) were used to check the mapping quality and gather information regarding GC content, insert size, coverage, percentage of aligned bases, general error rate, and duplication rate.

Accurate detection of variants is known to be problematic in DNA from FFPE tissues, as it often presents with fragmentations and changes that lead to sequence artifacts [16, 17]. These artifacts will likely present with strand orientation bias toward one of the directions; therefore, we processed the variant calling files (vcf) with SOBDetector, a software developed to detect strand orientation bias (SOB) in FFPE samples [18]. The BAM reads for all the variants were ultimately manually inspected, and a cross‐patient check was performed. Any somatic variants detected in samples from multiple patients were discarded, as this is an indication of an FFPE artifact.

For annotation and selection of the variants, we used a filtering cascade in VarSeq software (v.2.2.4, Golden Helix). We selected only for high‐quality and high confidence variants: they had to be covered by at least 25 reads and have a variant allele frequency (VAF) in reads greater than 0.1 (or 10%). A VAF of 0.1 is a commonly accepted threshold for detection of somatic variants in NGS [19]. The variants were further checked for strand orientation bias (acceptable SOB scores were equal or less than 0.8), and the calls had to have also a passed quality flag by the variant calling software. All variants located in multimapped regions, regions with short tandem repeats, or homopolymer regions were excluded. Regions with high background noise and low mean quality in BAM files (<20) were also excluded. For the specific detection of somatic variants in the *VHL* gene, all the BAM files were manually screened in the whole *VHL* gene region.

Regarding functionality, only variants affecting the protein sequence or with a clear effect on the splice site were included.

Prediction of functional deleteriousness was calculated using the Combined Annotation Dependent Depletion (CADD Scores 1.4) method with Phil's Read Editor score (PHRED) scaled scores [20]. The CADD algorithm includes conservation metrics, functional genomic data, exon‐intron boundaries, and protein functionality scores. A score equal or greater of 20 was used and it indicates that a variant is predicted to be among the 1% of the most deleterious substitutions. The database for nonsynonymous SNPs' functional prediction (dbNSFP) tool [21] was used as an alternative method to score deleteriousness; the tool incorporates six different algorithms for the evaluation of the functional impact of a variant, including SIFT, Polyphen, MutTaster, MutAssessor, FATHMM Pred, and FATHMM MKL. The Genome Aggregation Database (GnomAD) 4.2. exomes and genomes were used for population allelic frequencies.

### 2.4 Gene set overrepresentation analysis

Enrichment analysis using Gene Ontology (GO) terms allows to identify common pathways or a common function, topology, or process within a given set of genes. We used our list of genes with somatic variants and the Molecular Signature Database v2024.1.Hs (Human MSigDB) [22] to test for enrichment of GO terms and cell type signature sets (https://www.gsea‐msigdb.org/gsea/index.jsp). The latter is based on gene expression data characteristic for a specific cell type. We have used this database in conjunction with our list of genes with somatic variants. *P*‐values were calculated assuming hypergeometric distribution of data, and false discovery rates (FDR) adjusted for multiple comparisons were used. For interpretation and visualization of the GO enrichment results Cytoscape 3.10.2 with the ClueGO (v.2.5.10) plug‐in was used [23]. Nodes with *P*‐value <0.05 were used, and only node clusters are shown. ClueGO performs an ontology analysis showing nonredundant biological terms as clusters in a functionally grouped network.

### 2.5 Structural variant analysis

Somatic copy number variants (CNVs) were called using allele‐specific copy number analysis of tumors (ASCAT) algorithm with paired blood samples as reference [24]. ASCAT estimates allele copy numbers for all single nucleotide polymorphisms (SNPs) loci while simultaneously adjusting for tumor ploidy and nonaberrant cell mixture. The result is a genome‐wide copy number profile of gains, losses, loss of heterozygosities (LOHs), and other neutral events. The ASCAT tool specific version and code used can be found as well at the cgpwgs‐2.1.0 docker image (https://dockstore.org/containers/quay.io/wtsicgp/dockstore‐cgpwgs:2.1.0). We used Delly2 [25] for the specific discovery of complex genomic rearrangements, such as translocations, inversions, tandem duplications, or deletions, with somatic calling and matched tumor control sample analysis. Any possible overlap of CNVs between different samples was checked using Bedtools overlap.

## 3 Results

**Fig. 1:**
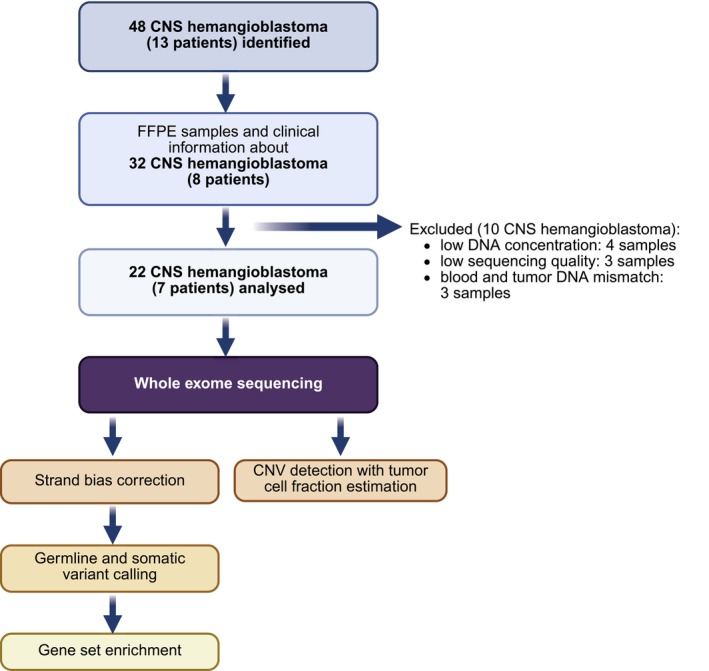
Overall study flowchart. Flowchart summarizing the sample collection and subsequent exclusion process, downstream sequencing, and data analysis. FFPE, formalin‐fixed paraffin‐embedded; CNS, central nervous system; CNV, copy number variant.

We analyzed somatic genetic changes occurring in hemangioblastomas from vHL patients to elucidate the molecular mechanisms underlying tumor development. We included patients with germline *VHL* variants known to result in loss of protein function (Table 1). In total, we performed WES on 22 CNS hemangioblastomas from seven patients, representing five unrelated families and four causative genotypes. These tumors had been resected over a period of 32 years (1981–2013). In Fig. 1 is shown a flowchart of the overall study plan and the performed analysis.

**Table 1:** Summary of WES results.

Patient	Family	Germline *VHL* variant	Tumors	No. of somatic point variants^a^	Somatic *VHL* variants	VAF (total read count)	CNV on *VHL* region
01	1	Del exon 2	CNS1	21	0		No solution
			CNS3	0	0		Normal
02	2	c.433C>T; p.Gln145Ter	CNS1	16	0		normal
			CNS2	8	c.488T>G; p.Leu163Arg	0.13 (188)	Normal
			CNS3	7	0		Heterozygous deletion
03	2	c.433C>T; p.Gln145Ter	CNS1	12	0		Heterozygous deletion
04	2	c.433C>T; p.Gln145Ter	CNS1	7	0		Heterozygous deletion
			CNS2	25	c.234T>G; p.Asn78Lys	0.19 (92)	Normal
			CNS3	2	0		No solution
05	3	c.481C>T; p.Arg161Ter	CNS1	1	0		No solution
			CNS2	2	c.477_478insCA; p.Glu160Glnfs*11	0.08 (252)	Normal
			CNS3	3	c.181delC; Val62Cysfs*5	0.14 (99)	Normal
			CNS4	1	0		Normal
			CNS5	4	0		Heterozygous deletion
06	4	del exon 2	CNS1	19	c.454dupA; p.Thr152Asnfs*22	0.27 (22)	Normal
			CNS3	13	0		Heterozygous deletion
			CNS4	6	0		Normal
			CNS6	1	0		Normal
			CNS7	0	0		Normal
			CNS9	12	0		Heterozygous deletion
08	5	del exon 3	CNS1	3	c.634_635insGATGGAA; p.Gly212Glufs*46	0.14 (79)	Duplication without LOH
			CNS2	7	c.462delA; p.Val155Cysfs*4	0.11 (76)	Heterozygous deletion
Total				170			

a The numbers refer to the total number of single nucleotide somatic variants identified, including those identified in the *VHL* gene only by manual screening.

Detailed clinical information of the patients and the tumors is provided in Table S1. In brief, the cohort included three female and four male patients with the median age of 21 at diagnosis of the first vHL tumor (range 11–35 years). A total of 22 tumors were analyzed: 17 cerebellar, three spinal, and two brain stem. Most tumors (17 of 22) were cyst‐associated at resection. The median time from diagnosis to resection was 23 months (range 0–128). Imaging data were available for 16 tumors: six showed continuous growth, three were stable in size, and seven displayed a stuttering growth pattern (periods of no growth followed by rapid growth), typically preceding symptom onset and surgery and consistent with previous reports [1, 5].

### 3.1 Mutational landscape of hemangioblastomas from vHL patients

An overview of the samples and a summary of the analysis is reported in Table 1, while details on the sequencing output are listed in Table S2. We successfully analyzed between one and six tumors from each patient. We found a second‐hit *VHL* variant in more than half (59%, 13/22) of the hemangioblastomas; all of these tumors (100%, 13/13) carried a somatic variant that would presumably lead to bi‐allelic inactivation of *VHL*. Of these, eight samples had a *VHL* copy number alteration and seven samples had a small indel or a missense variation. Two samples had both a CNV and an indel variant. All indel *VHL* variants resulted in frameshifts leading to downstream stop codons, thus altering heavily the protein structure and functionality. Only two *VHL* somatic variants were missense: c.234T>G; p.Asn78Lys (exon 1), and c.488 T>G; p.Leu163Arg (exon 3). Both of these are predicted to be deleterious; they are both situated in a mutational hotspot and are absent from GnomAD 4.2. The amino acid changes, p.Leu163Arg, have been previously described in relation to vHL disease as a germline variant in a family with pheochromocytomas [26], while p.Asn78Lys has not been reported before. Figures illustrating sequencing coverage read alignments from the BAM files for all identified second‐hit *VHL* variants are provided in Figs S1–S10.

**Fig. 2:**
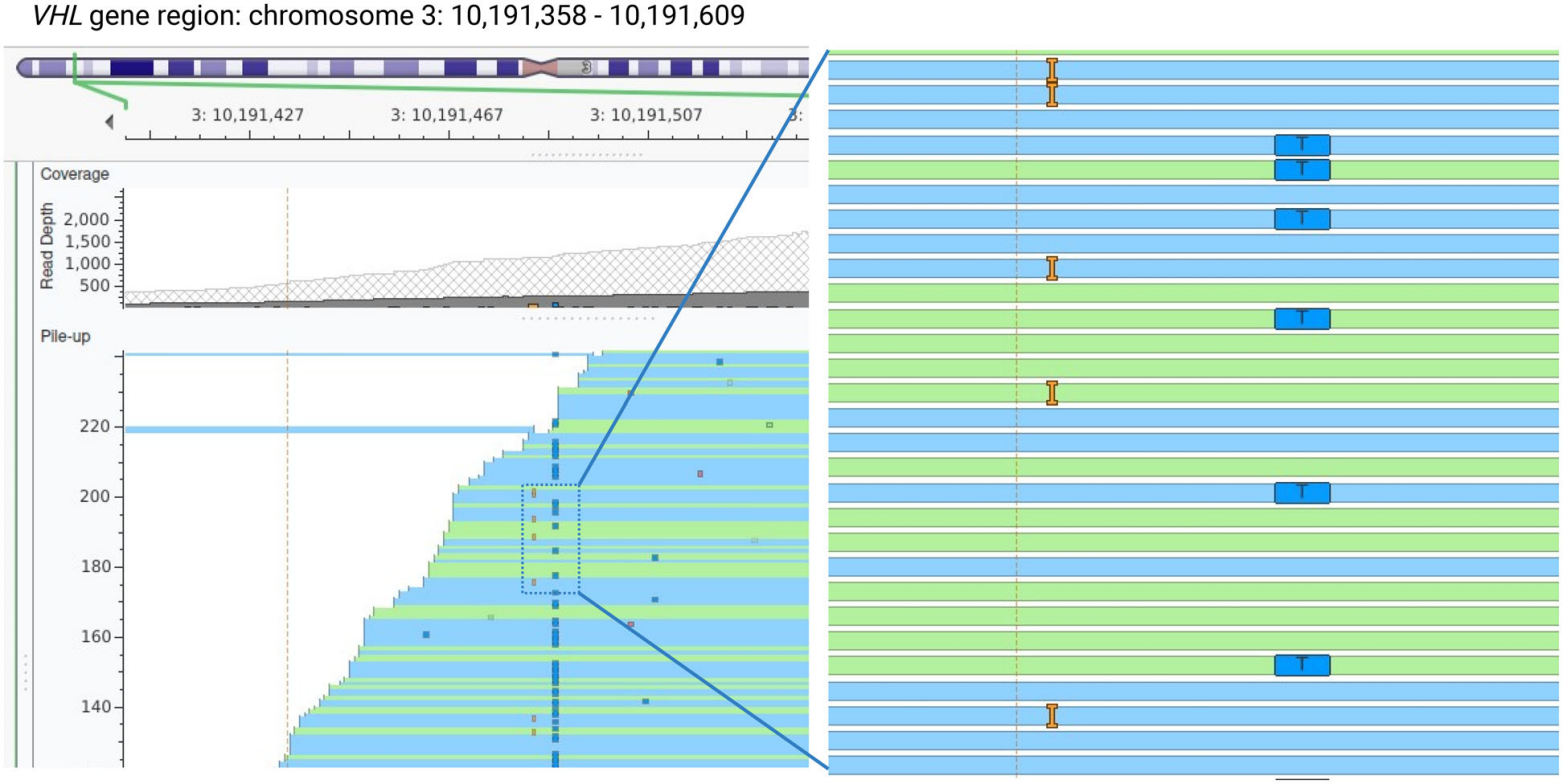
Germline and second‐hit *VHL* variants in sample CNS2 from patient 05 are located on different alleles generating loss of heterozygosity. The binary alignment map (BAM) file for sample CNS2 from patient 05 is shown as visualized in the VarSeq software, depicting the sequencing reads over the *VHL* exon 2 region. The reads are in green or blue color depending on the left or right orientation. The bigger picture is an enlarged view over the position of the variants. On the top, the coordinates on chromosome 3 are visualized. Below on the y‐axis, the overall coverage of the sequence can be observed (read depth). The *VHL* germline variant c.481C>T (p.Arg161Ter) is shown as a blue rectangle. The second‐hit variant is an insertion, c.477_478insCA, which generates a frameshift (p.Glu160Glnfs*11), and it is represented by an orange mark. The total read depth over the region was 252, while the proportion of reads with the variant allele was 0.08%. Only a small percentage of the overall reads is affected by the variant, as generally observable for somatic variants; however, the two variants never occur on the same read, thus visualizing the bi‐allelic inactivation of *VHL*.

In one tumor (CNS2 from patient 05), the *VHL* somatic variant c.477_478insCA was detected positioned in close proximity to the germline variant (c.481C>T; p.Arg161Ter). Figure 2 shows the sequencing coverage and the position of the two variants, confirming second‐hit occurrence, with the effect of complete inactivation of *VHL* in the tumor. Tumor percentage could be estimated to be 14.5%, based on the ratio between the number of reads carrying the wild‐type and the somatic variant.

The overall somatic mutational burden in vHL‐related CNS hemangioblastomas was relatively low. Across the 22 samples, we identified 170 unique somatic variants affecting 162 genes (a complete list is provided in Table S3). The two variant calling software used for single nucleotide polymorphisms (Mutect2 and CaVEMan) were in agreement for 36 variants, with Mutect2 detecting more variants than CaVEMan. PinDel did not detect any indel variants (Table S4). Therefore, we used data from both Mutect2 and CaVEMan and manually validated all the variants by visual inspection of the BAM files.

We found on average 7.7 (0–25) somatic variants (SNVs and indels) per tumor, with *VHL* being most frequently mutated gene, as it is the only gene, besides *NIN* and *TTN*, that had somatic variants in more than one sample.

In two tumor samples, no somatic variants were detected. These samples originated from two unrelated patients, both carrying a germline exon 2 deletion in *VHL*. The absence of detectable variants may reflect technical limitations, such as low tumor cell proportion in the samples. By contrast, other samples carrying the exon 2 deletion along with additional somatic *VHL* variants tended to exhibit a higher mutational burden. Notably, all tumors with the germline exon 2 deletion and multiple somatic variants (*N* = 6) were resected very shortly after initial diagnosis (between 2 days and 18 months; median 2 weeks), suggesting a potentially more aggressive tumor growth.

Tumors harboring the p.Arg161Ter germline variant exhibited few somatic variants and the lowest mutational burden. In contrast, the tumor with absolutely the most somatic alterations (tumor CNS2 from patient 04) carried the germline *VHL* variant p.Gln145Ter as well as the somatic *VHL* variant c.234T>G, p.Asn78Lys and a total of 25 additional variants (Table 1). This tumor was described to have developed associated cyst formation just prior to surgical resection; the cyst was characterized by multiple cyst chambers containing multiple solid elements.

### 3.2 Gene ontology analysis

**Fig. 3:**
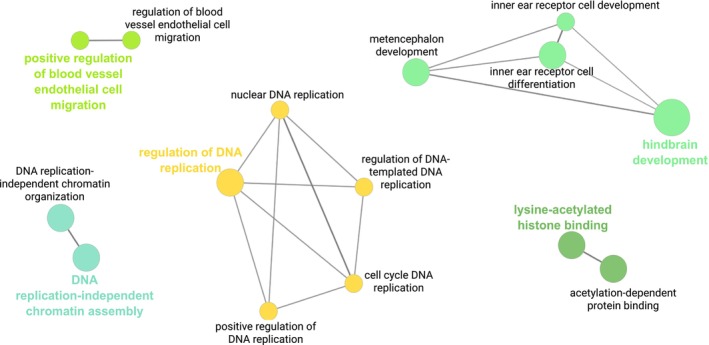
Gene Ontology analysis. The 162 genes identified as bearing somatic variants in 22 hemangioblastomas were further analyzed for gene set overrepresentation of Gene Ontology (GO) gene sets. For visualization, Cytoscape and ClueGO software tools were used with all three GO ontologies as reference set. The clusters show the most represented gene sets affected by somatic variants. The node sizes show the significance of the term with the biggest being the most significant. The node color represents GO terms within the same larger GO group.

**Fig. 4:**
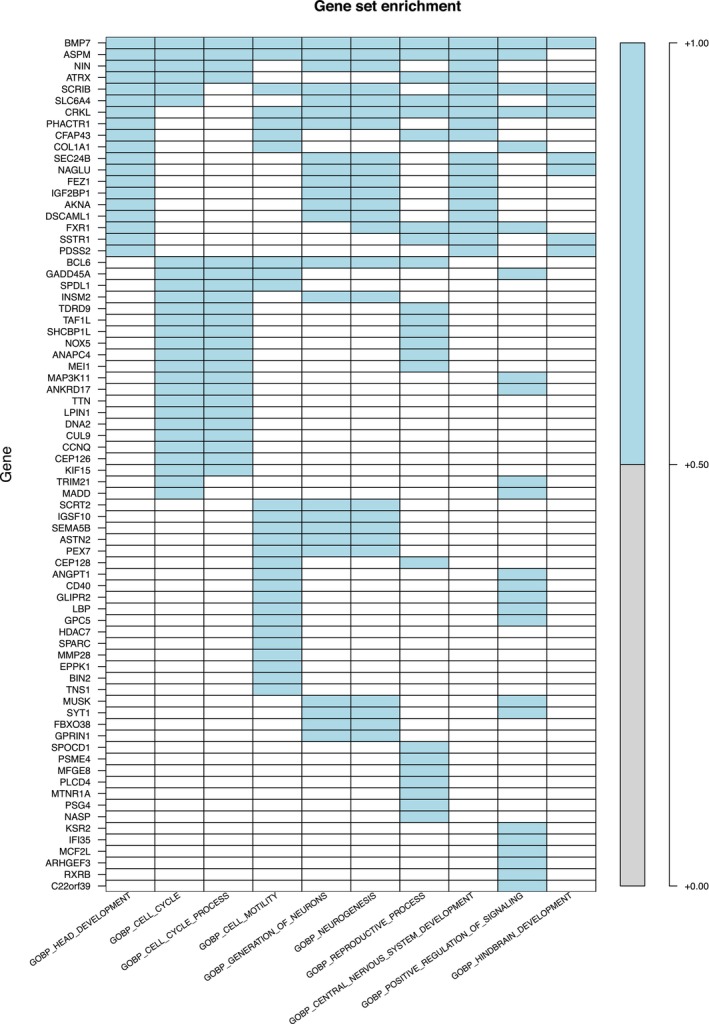
Gene ontology overrepresentation analysis of somatic variants found in CNS hemangioblastomas. The 162 genes obtained from the somatic variant analysis of the 22 hemangioblastomas were subsequently analyzed with the Human MSigDB tool to check whether there are overrepresented gene sets. In the figure, the 10 most significantly overrepresented gene sets (FDR or p‐values after correction according to Benjamini and Hochberg ≤1.32 × 10^−4^) within the Gene Ontology (GO) database are listed horizontally on the matrix, while the gene names are listed vertically. The whole list of pathways is supplemented in Table S5. GOBP, Gene Ontology Biological Process.

All genes harboring somatic variants were analyzed for gene set enrichment with Human MSigDB. Significantly enriched gene sets are listed in Table S5. In total, 80 gene sets were identified as significantly enriched across the GO categories of biological process (BP), molecular function (MF), and cellular component (CC). To facilitate the interpretation, gene sets were grouped based on similarity using ClueGO. The results are presented in Fig. 3 highlighting the most prominently represented gene sets. The biggest groups are genes related to the regulation of DNA replication and hindbrain development. Regulation of blood vessel endothelial migration is also one of the most represented gene sets together with histone binding and chromatin assembly gene sets, showing that genes involved in these processes are often affected by somatic changes in hemangioblastoma. Analysis of the 10 most significantly overrepresented GO terms (Fig. 4), in conjunction with genes harboring somatic mutations, yielded results consistent with those obtained using ClueGO. The top 10 terms were predominantly associated with CNS development and the cell cycle. Notably, the *VHL* gene was not included in any of the 10 most overrepresented gene sets.

### 3.3 Cell‐type signatures analysis

**Fig. 5:**
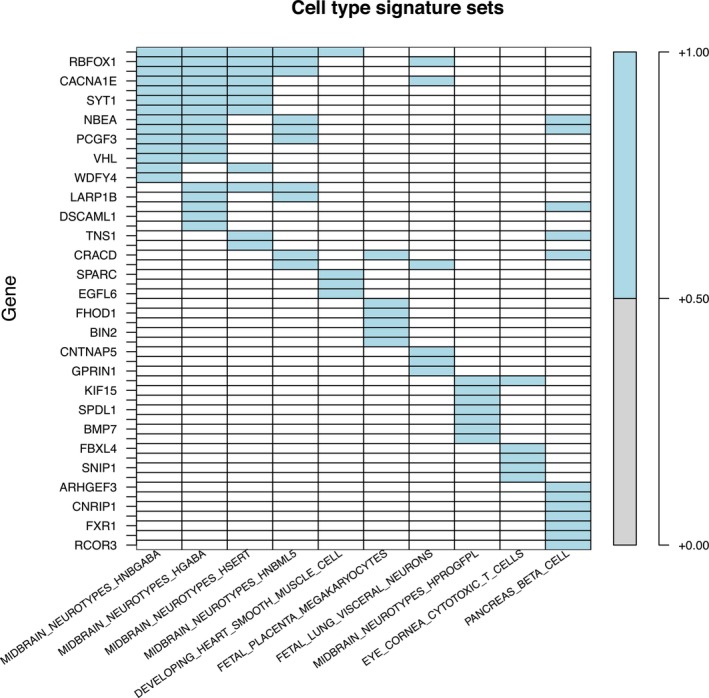
Cell‐type signatures overrepresentation analysis. The 162 genes identified as bearing somatic variants in the 22 hemangioblastomas were further analyzed for overrepresentation of cell types using the cell‐type signature gene sets with Human MSigDB tool. The ten most significantly overrepresented categories (FDR or p‐values after correction according to Benjamini and Hochberg ≤1.88 × 10^−2^) are included in the figure. Cell type categories showing enrichment of genes are listed horizontally, while the gene names are listed vertically. The complete list of cell types is supplemented in Table S6.

We analyzed our list of genes harboring somatic variants to determine whether certain cell type–specific genes were overrepresented. Among the 10 most significantly enriched gene sets, many were characteristic of neuronal cells. In particular, we identified human embryonic midbrain neuronal cells (Table 2 and Fig. 5) as defined by La Manno et al. [27], including gamma aminobutyric acid‐ (GABA)‐ergic and serotonergic neurons, mediolateral neuroblasts, and neuronal progenitor cells of the lateral floorplate. *VHL* is included in the gene sets defining the GABAergic neurotypes. As this may cause intrinsic bias, we have repeated the analysis without the *VHL* gene in the list, which resulted in only minimal changes to the cell type categories, as shown in Table S6. Both lists are provided in Table S6.

**Table 2:** Gene set enrichment analysis: cell type signatures.

Gene set name	Genes in overlap (k)	Strength (k/K)	*P*‐value	FDR q‐value
MANNO_MIDBRAIN_NEUROTYPES_HNBGABA	14	0.0199	5.53E‐7	3.73E‐4
MANNO_MIDBRAIN_NEUROTYPES_HGABA	17	0.0154	1.19E‐6	3.73E‐4
MANNO_MIDBRAIN_NEUROTYPES_HSERT	11	0.0244	1.35E‐6	3.73E‐4
MANNO_MIDBRAIN_NEUROTYPES_HNBML5	10	0.0215	1.26E‐5	2.61E‐3
CUI_DEVELOPING_HEART_SMOOTH_MUSCLE_CELL	4	0.0833	3.4E‐5	5.65E‐3
DESCARTES_FETAL_PLACENTA_MEGAKARYOCYTES	6	0.0296	1.29E‐4	1.78E‐2
DESCARTES_FETAL_LUNG_VISCERAL_NEURONS	6	0.0283	1.67E‐4	1.98E‐2
MANNO_MIDBRAIN_NEUROTYPES_HPROGFPL	7	0.0217	2.43E‐4	2.17E‐2
GAUTAM_EYE_CORNEA_CYTOTOXIC_T_CELLS	5	0.0338	2.63E‐4	2.17E‐2
MURARO_PANCREAS_BETA_CELL	12	0.0127	2.81E‐4	2.17E‐2

Top ten enriched cell‐type signatures using all the genes with somatic variants found in our study. The set name is named after the author describing the molecular signature (https://www.gsea‐msigdb.org). For neurotype gene sets H stands for human, NB for neuroblast, GABA for GABAergic (HNBGABA, HGABA), SERT for serotonergic (HSERT), HNBML5 for mediolateral neuroblasts, and HPROGFPL for neuronal progenitors of the lateral floorplate. Genes in overlap (k) are the number of genes from our results found in the specific group; strength (k/K) is the ratio between the genes in overlap and total number of genes in the category; a *P*‐value and a false discovery rate (FDR) *q*‐value calculated to correct for multiple testing.

### 3.4 Copy number analysis

**Fig. 6:**
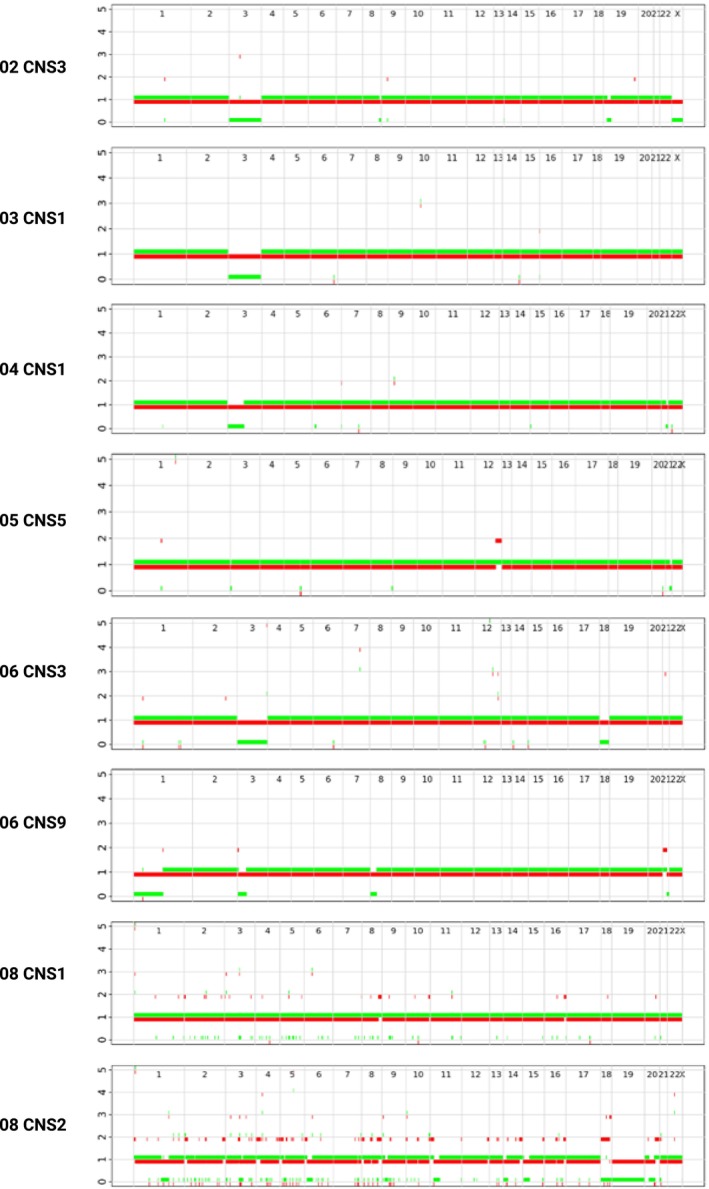
ASCAT profiles of samples with an alteration of copy number in the *VHL* region. Whole genome Allele‐Specific Copy Number Analysis of Tumors (ASCAT) profiles were calculated for each of the 22 tumors and a graphical representation is used to show the global ploidy status. In the figure are shown those samples where allelic loss or other events were detected on chromosome 3 in the *VHL* gene region. ASCAT takes into consideration a putative aberrant cell fraction and tumor ploidy values to calculate a genomic profile of the tumor. The profile displays the calculated copy number of all loci (on the y‐axis) for all the chromosomes (x‐axis). The green allele is always the allele with the lowest copy number, while the red one is the allele with the highest copy number.

Besides analysis of single nucleotide variants and small indels, we also performed copy number and chromosomal rearrangement analysis. We observed that CNVs of the region containing the *VHL* gene were the most frequently occurring event. Ploidy profiles for the tumor samples with a detectable copy number alteration in the *VHL* region are shown in Fig. 6 and Table 3 (while in Fig. S11 data for all the samples are provided). Copy number alterations in the *VHL* gene region were detected in 8 tumors using the ASCAT algorithm. In seven out of the eight cases, we detected LOH in a chromosomal region encompassing the *VHL* gene, while a single duplication event was observed in sample 08‐CNS2. The latter was considered functionally neutral, as no concomitant LOH was detected. Allelic loss occurred over either the shorter regions of the 3p arm (05‐CNS5 and 08‐CNS2) or longer regions (04‐CNS1 and 06‐CNS9), and in three tumors, the entire chromosome 3 was lost (02‐CNS3, 03‐CNS1, and 06‐CNS3). In three samples the analysis was inconclusive or yielded no solution, typically due to an undeterminable tumor fraction or inability to calculate allelic frequencies. These issues generally result from insufficient tumor content or inadequate sequencing depth.

**Table 3:** Results from the CNV analysis of the *VHL* region.

Sample	Gene	Annotation	Chromosome	Start	End	Normal ploidy	Normal minor	Tumor ploidy	Tumor minor
02‐CNS3	*VHL*	LOH	3	60.197	49.943.200	2	1	1	0
03‐CNS1	*VHL*	LOH	3	60.197	197.908.615	2	1	1	0
04‐CNS1	*VHL*	LOH	3	60.197	89.523.038	2	1	1	0
05‐CNS5	*VHL*	LOH	3	60.197	10.329.377	2	1	1	0
06‐ CNS3	*VHL*	LOH	3	60.197	195.447.011	2	1	1	0
06‐CNS9	*VHL*	LOH	3	9.955.070	48.694.147	2	1	1	0
08‐CNS1	*VHL*	Duplication	3	10.669.760	10.689.671	2	1	5	2
08‐CNS2	*VHL*	LOH	3	7.153.339	10.681.351	2	1	1	0

Samples presenting with an alteration of the *VHL* gene region (chromosomal coordinates 3: 7.153.338–10.681.351) as calculated with the ASCAT algorithm. In seven samples, allelic loss was detected through a deletion event in the aberrant cell fraction of the sample (indicated as LOH). In one sample the tumor ploidy was over 2n (08‐CNS1), indicating a duplication event has occurred. The event is considered neutral as both alleles are still present. The start and the end are chromosome 3 coordinates. When calculating the tumor ploidy across the genome, ASCAT takes into consideration that there is a fraction of tumor cells and a normal cell fraction. For both of these fractions, the ploidy is calculated and reported (normal ploidy and tumor ploidy). Normal and tumor minor indicate the number of copies of the least prevalent allele in the respective fraction.

**Fig. 7:**
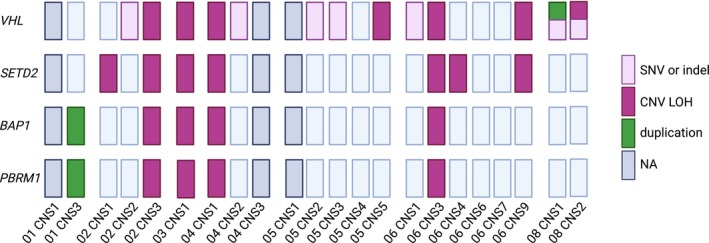
High frequency of copy number alterations in regions containing *VHL*, *SETD2*, *BAP1*, and *PBRM1* genes in familial hemangioblastoma. Summarized results from the whole exome sequencing of 22 hemangioblastoma, showing somatic single nucleotide (SNV), indel variants, and copy number variants (CNVs) with loss of heterozygosity (LOH) in *VHL* and an additional three (*SETD2*, *BAP1*, and *PBRM1*) genes also residing on 3p. These genes are frequently found codeleted with *VHL* in clear cell renal carcinoma (ccRCC). Each set of blocks is a patient in the cohort; light red indicates presence of somatic single nucleotide variants or small indels, full red indicates deletion of larger chromosome 3 regions with loss of heterozygosity, and bright green indicates a copy‐neutral duplication event; NA indicates that the CNV analysis was inconclusive.

When looking for other copy number alterations occurring in multiple samples, we did not detect any other recurrent signal. As partial or total deletion of 3p was the most frequent event, we checked the status of other three genes residing in 3p, which have been reported often to be codeleted with *VHL* in clear cell renal carcinoma (ccRCC). These are namely *PBRM1*, *BAP1*, and *SETD2*. In our data, these three genes were also often deleted or co‐deleted with *VHL* (Fig. 7). Two samples (02‐CNS1 and 06‐CNS4) had no somatic *VHL* alteration, but they had loss of a *SETD2* allele. Additionally, sample 06‐CNS9 presented with LOH in both *VHL* and *SETD2*, but not in *BAP1* and *PBRM1*.

We did not identify any complex chromosomal rearrangements such as inversion, insertions, fusions, or tandem duplications in our samples.

## 4 Discussion

The mechanisms underlying tumor development in vHL disease remain incompletely understood, and systematic approaches with a broad perspective are essential to advance our knowledge of the genetic events that drive the progression from early precursors to fully developed tumors. To date, our study represents the largest study based on WES of familial hemangioblastoma. We comprehensively investigated SNVs, indels, CNVs, and large‐scale chromosomal rearrangements at the exome level.

We report a low overall mutational burden, but a highly prevalent *VHL* inactivation, with inactivating second‐hit variants identified in more than half (59%, 13/22) of the tumors. The *VHL* gene was most frequently affected by somatic copy number alterations (36%, 8/22), followed by small indels and single nucleotide variants occurring only a bit less frequently (32%, 7/22). Somatic missense *VHL* variants were seen only in two tumors. The variant p.Asn163Lys has been previously described as a germline variant in a family with pheochromocytomas [26], while the variant p.Asn78Lys has not previously been described in vHL. However, other variants at protein position 78 have been described in vHL, suggesting that this is an important amino acid residue for the protein functionality. In our data, the p.Asn78Lys somatic variant was detected in a sample harboring the p.Gln145Ter germline variant; this tumor was the one with most somatic variants (25 in total).

We observed the hemangioblastoma exome to be devoid of a large burden of alterations characterizing other types of tumors, probably reflecting its ‘benign’ designation. Only a few samples had a more complex genomic profile, potentially reflecting the resection of multiple co‐localized tumors. Furthermore, each tumor harbored a distinct set of somatic variants, with no overlap observed between samples from the same patient. This lack of shared clonality indicates that individual lesions arise independently rather than from a common ancestral clone. Our findings are consistent with previous reports on both familial and sporadic hemangioblastomas [28, 29, 30, 31, 32], and in support of hemangioblastomas being always primary tumors.

In total, we found somatic variants in 162 genes. A gene set enrichment analysis revealed that there is a significant overrepresentation of genes related to DNA replication, hindbrain development, blood vessel cell migration, and histone binding. This is an indication that somatic variants in these sets of genes predispose to hemangioblastoma tumor formation in the CNS. We also found an overrepresentation of somatic variants in genes that are known to be expressed in GABAergic and serotonergic neurons, with *VHL* itself being part of gene signatures associated with GABAergic neurons. Analysis of our catalog of affected genes further revealed that many of the enriched cell types corresponded to neuroblasts and neuronal progenitors. These findings suggest that cells with transcriptional programs characteristic of undifferentiated neuronal cells may be particularly susceptible to hemangioblastoma formation. This is consistent with previous reports describing neoplastic ‘stromal’ cells in hemangioblastoma expressing markers typically associated with embryonic progenitor cells [33]. Together, these observations support the hypothesis that hemangioblastoma may originate from a population of progenitor cells with impaired differentiation capacity, providing a possible cellular explanation for their distinct biology and topology. Since we do not measure actual gene expression, but we test for enrichment only using the list of genes with somatic variants, our findings need confirmation in future studies where somatic variant detection is coupled with detection of gene expression.

Overall, recurrent somatic alterations were observed at the *VHL* locus, including single nucleotide variants, small indels, hemizygous loss of the *VHL* locus, loss of the 3p arm or the entire chromosome 3, and chromosome 3 duplication, consistent with previous studies of sporadic hemangioblastoma [11, 32]. Deletion size in chromosome 3 was previously reported to be very variable in vHL, but not correlated with the germline variant, affected organ, or type and behavior of the tumor [11].

Although *VHL* LOH was frequent in our dataset, not all tumors harbored a detectable second‐hit variant in the *VHL* gene. This may reflect technical limitations, such as low tumor content or sample type; for example, but it also suggests that additional genes may be involved. Previous investigations reported that *VHL* inactivation alone is not sufficient for the development of vHL‐related tumors [11, 31, 34], and other genetic targets have been researched including, for example, *HNF1B* [35] and *PLAGL1* (or *ZAC1*) [36, 37]. We did not detect any copy number or single nucleotide variants in previously reported genes, nor did we find any recurrent variants in other genes. However, we did find a somatic variant in *KDM5C* (c.4159C>T), which is the most frequently mutated gene outside 3p in ccRCC [38].

In ccRCC, co‐deletions and single nucleotide variants affecting *VHL* together with one or more additional tumor suppressor genes also located on the short arm of chromosome 3 (*PBRM1*, *BAP1*, and *SETD2*) have been well‐documented [38, 39, 40, 41]. Although we did not identify somatic single nucleotide variants in these genes, we frequently observed allelic loss affecting one or more of them. Notably, two samples carried a heterozygous deletion including *SETD2* but not *VHL*. These findings suggest that, in addition to their established role in ccRCC, these genes may also contribute to hemangioblastoma tumorigenesis, providing a basis for further investigation.

### 4.1 Strengths, limitations, and future perspectives

The major strength of this study is that it provides whole exome investigations on the largest set of familiar hemangioblastoma samples reported to date, with clinical characteristics and tumor growth patterns included. In most cases, we successfully collected and analyzed multiple tumors per patient. Such analyses are challenging, in part due to the time required for sample collection and the use of FFPE tissue. While successful detection of somatic variants in FFPE samples represents an achievement, it also constitutes a limitation, as nucleic acids in FFPE tissue are often fragmented and prone to artifacts, complicating the analysis. To address this, we used exome sequencing to achieve a higher sequencing depth and bioinformatic tools, such as a strand‐bias detector, to distinguish real variants from FFPE‐specific false positives, as the latter will typically present strand bias. We also set the minimum VAF threshold to 0.1, under which we considered the risk of false positives to outweigh the potential gain in sensitivity. Ultimately, we conducted manual inspection of BAM reads and performed cross‐checks across samples from different patients. These combined strategies, from high‐depth sequencing, bioinformatics quality checks, to manual quality control, allowed us to confidently distinguish real variants from artifacts in our FFPE samples.

Additional limitations include incomplete gene coverage by the commercial exome kit—previously reported genes such as *HNF1B* were not included. Finally, although this represents the largest genome‐wide investigation of familial hemangioblastoma to date, the overall number of samples remains limited, making phenotype–genotype correlation very difficult. While this study shows the feasibility of somatic variant analysis in FFPE samples of vHL hemangioblastoma, future studies are needed to increase the number of samples for reliable assessments with clinical phenotypes.

## 5 Conclusions

Our findings indicate that vHL‐related familial hemangioblastomas are characterized by instability of the *VHL* gene and extended regions of chromosome 3, reinforcing the central role of this locus in tumor initiation. Despite the overall low somatic mutation burden, the significant enrichment of alterations in genes associated with cell cycle regulation and neurogenesis suggests that selective pressures act on pathways critical for proliferation and neuronal differentiation in certain cell types (GABAergic and serotonergic neuronal cell types). These results support a model in which *VHL* loss establishes the permissive background for tumor formation, while secondary mutations in specific developmental and cell cycle‐related genes contribute to tumor progression.

## Conflict of interest

MLMB is presently employed by H. Lundbeck A/S. The employment is unrelated to the present work, which was conducted while MLMB was employed at the University of Copenhagen. The other authors declare no conflicts of interest.

## Author contributions

MD performed part of the bioinformatic data analysis, manually curated the somatic variants, organized the data, and wrote the manuscript. AN has participated in the supervision of the project and the discussions during sample collection and patient inclusion phases. LvBA performed part of the data analysis. LBO, as the leader of the national Danish database of vHL patients, has helped with the definition of the project and selection of the patients. MLMB defined the project, acquired the funding, recruited the patients, provided the samples, and analyzed and evaluated the clinical data. MT supervised the project including data acquirement and analysis, and the writing of the manuscript. All authors discussed the results and commented on the manuscript.

## Supporting information


**Fig. S1.** Binary alignment map showing reads over the somatic variant detected in patient 02, CNS2, *VHL*: c.488T>G; p.Leu163Arg.
**Fig. S2.** Binary alignment map showing reads over the somatic variant detected in patient 04, CNS2, *VHL*: c.234T>G; p.Asn78Lys.
**Fig. S3.** Binary alignment map showing reads over the variants detected in patient 05, CNS2, somatic *VHL*: c.477_478insCA; p.Glu160Glnfs*11 and germline *VHL*: c.481C>T; p.Arg161Ter.
**Fig. S4.** Binary alignment map showing reads over the *VHL* variants detected in patient 05, CNS2, larger view if the region.
**Fig. S5.** Binary alignment map showing reads over the somatic variant in patient 05, CNS3, *VHL*: c.181delC; Val62Cysfs*5.
**Fig. S6.** Binary alignment map showing reads over the somatic variant in patient 06, CNS1, *VHL*: c.454dupA; p.Thr152Asnfs*22 somatic insertion.
**Fig. S7.** Binary alignment map showing reads over the somatic variant in patient 08, CNS1, *VHL*: c.634_635insGATGGAA; p.Gly212Glufs*46.
**Fig. S8.** Binary alignment map showing reads over the somatic variant in patient 08, CNS1, larger view of the region.
**Fig. S9.** Binary alignment map showing reads over the somatic variant in patient 08, CNS2, *VHL*: c.462delA; p.Val155Cysfs*4 somatic deletion.
**Fig. S10.** Binary alignment map showing reads over the somatic variant in patient 08, CNS2, larger view of the region.
**Fig. S11.** Whole genome profiles of all 22 samples: results from the ASCAT analysis.
**Table S1.** Clinical characteristics of included vHL patients and CNS hemangioblastomas.

**Table S2.** NGS sequencing metrics.

**Table S3.** List of all single nucleotide and small indels somatic variants detected.
**Table S4.** Number of variants as identified by different variant callers.

**Table S5.** 100 most significantly enriched GO terms.
**Table S6.** 100 most significantly enriched cell types signature sets.

**Table S7.** Tabular results of ASCAT analysis.

## Data Availability

In order to comply with the ethical approval, all anonymized data supporting the findings of this study are disclosed within the main article and its supplementary data, including the complete list of somatic variants detected, single nucleotide variants, indels, and copy number variations. Raw sequencing data are not available for broad data sharing because of the ethical and legal restrictions on data usage in Denmark. The underlying code for this study is available in: Mutect2 is found at GATK, accessed via the link https://gatk.broadinstitute.org/hc/en‐us/; ASCAT, Pindel, and CaVEMan tools specific versions and code used can be found at the cgpwgs‐2.1.0 docker image (https://dockstore.org/containers/quay.io/wtsicgp/dockstore‐cgpwgs:2.1.0). Delly tools code can be found at https://hub.docker.com/r/dellytools/delly/.
