# Supplementary material for: Somatic mutational landscape in von Hippel–Lindau familial hemangioblastoma
Source: Mol Oncol. 2026 Mar 23:10.1002/1878-0261.70228. Online ahead of print. doi: 10.1002/1878-0261.70228 (PMC13398559; doi:10.1002/1878-0261.70228)

**Table S1. Clinical characteristics of included vHL patients and CNS hemangioblastomas**

| **Patient no.** | **VHL family** | **Sex**  **(F: Female/ M:Male)** | **Germline *VHL* variant** | **Age at first vHL tumor diagnosis (years)** | **Tumors included in WES analysis** | | | | |
| --- | --- | --- | --- | --- | --- | --- | --- | --- | --- |
|  |  |  |  |  | **Tumor number** | **Tumor type and location** | **Time from first tumor diagnosis to resection (diagnosed due to symptoms (S) or asymptomatic (A) at diagnosis due to vHL surveillance)** | **Cyst formation associated with tumor** | **Growth characteristics** |
| **01** | 1 | F | c.341-? - 463+?del (del exon 2) | 19 | CNS1 | **Brainstem hb** (level of foramen magnum) | 2 days (S) | No | **No information on growth pattern** (no imaging before resection). |
|  |  |  |  |  | CNS3 | **Brainstem hb** (level of C2) | 54 months (A) | Yes (not at diagnosis, cyst formation prior to resection) | **Stuttering growth pattern*^1^:** No growth for first 49 months, sudden and rapid growth with doubling of tumor size and cyst formation within 5 months. |
| **02** | 2 | F | c.433C>T (p.Gln145Ter) | 35 | CNS1 | **Cerebellar hb** (right cerebellar hemisphere) | 2.5 months (S) | Yes | **No information on growth pattern** (no imaging before resection). |
|  |  |  |  |  | CNS2 | **Cerebellar hb** (right cerebellar hemisphere) | 116 months (A) | Yes | **Steady growth of** tumor over about 104 months. Cyst reduced at operation 1 month after diagnosis, steady reformation until resection. |
|  |  |  |  |  | CNS3 | **Cerebellar hb** (left cerebellar hemisphere) | 119 months (A) | Yes (not at diagnosis, cyst formation prior to resection) | **Stuttering growth pattern*^1^:** No growth for about 14 months after diagnosis. Steady growth for 58 months. No growth for the next approximately 36 months. Growth and cyst formation during the last 6 months prior to resection. |
| **03** | 2 | F | c.433C>T (p.Gln145Ter) | 21 | CNS1 | **Cerebellar hb** (right cerebellar hemisphere) | 95 months (S) | Yes (not at diagnosis, cyst formation prior to resection) | **Stuttering growth pattern*^1^:** No tumor growth and cyst formation until 1 month prior to resection: tumor growth and rapid cyst formation. |
| **04** | 2 | M | c.433C>T (p.Gln145Ter) | 22 | CNS1 | **Cerebellar hb** (left cerebellar hemisphere) | 43 months (S)*^2^ | No | **Steady growth** over 43 months. |
|  |  |  |  |  | CNS2 | **Cerebellar hb** (right cerebellar hemisphere) | 69 months (S) *^2^ | Yes (not at diagnosis, cyst formation prior to resection) | **Stable tumor size, cyst formation and development of multiple tumor elements:** Single solid tumor stable for about 42 months after diagnosis, thereafter cyst formation. Stable size of cyst, but multiple cyst chambers with multiple solid elements just prior to resection. |
|  |  |  |  |  | CNS3 | **Cerebellar hb** (right cerebellar hemisphere) | 128 months (S) | Yes (not at diagnosis, cyst formation prior to resection) | **Stuttering growth pattern*^1^:** No growth about 72 months after diagnosis. Thereafter 36 month period with steady growth of tumor, then no further growth. Cyst formation about 27 months after diagnosis, cyst punctured. New cyst development 72 months after initial diagnosis. Stable cyst size until 6 months prior to resection, when the cyst diameter doubled in size. |
| **05** | 3 | M | c.481C>T (p.Arg161Ter) | 11 | CNS1 | **Cerebellar hb** (cerebellar vermis) | 1 week (S) | Yes | **No information on growth pattern** (no further imaging before resection). |
|  |  |  |  |  | CNS2 | **Cerebellar hb** (left cerebellar tonsil) | 28 months (A) | No | **Stuttering growth pattern*^1^:** No tumor growth from diagnosis until about 2 months prior to removal. |
|  |  |  |  |  | CNS3 | **Cerebellar hb** (right cerebellar tonsil) | 28 months (A) | Yes (not at diagnosis, cyst formation prior to resection) | **Stable tumor size, cyst formation:** cyst formation about 2 months prior to resection. |
|  |  |  |  |  | CNS4 | **Cerebellar hb** (left cerebellar hemisphere) | 2 months (A) | Yes | **Steady growth** over 2 months. |
|  |  |  |  |  | CNS5 | **Spinal hb** (level of L1) | 34 months (A) | No | **Stable tumor size** from diagnosis to resection |
| **06** | 4 | M | c.341-? - 463+?del (del exon 2) | 13 | CNS1 | **Cerebellar hb** (left cerebellar hemisphere) | 1 week (S) | Yes | **No information on growth pattern** (no further imaging before resection). |
|  |  |  |  |  | CNS3 | **Cerebellar hb** (right cerebellar hemisphere) | 2 weeks (S) | Yes | **No information on growth pattern** (no further imaging before resection). |
|  |  |  |  |  | CNS4 | **Cerebellar hb** (right cerebellar hemisphere) | 2 weeks (S) | Yes | **No information on growth pattern** (no further imaging before resection). |
|  |  |  |  |  | CNS6 | **Cerebellar hb** (right cerebellar hemisphere) | 18 months (A) | No | **Steady growth** over 18 months. |
|  |  |  |  |  | CNS7 | **Cerebellar hb** (right cerebellar hemisphere) | 18 months (A) | No | **Steady growth** over 18 months. |
|  |  |  |  |  | CNS9 | **Cerebellar hb** (left cerebellar hemisphere) | 18 months (A) | Yes | **Stuttering growth pattern*^1^:** No tumor growth from diagnosis until about 4 months prior to removal. |
| **08** | 5 | M | c.464-?_642+ ?del (del exon 3) | 32 | CNS1 | **Spinal hb** (level of T11) | 76 months (A) | Yes | **Stuttering growth pattern*^1^:** No growth for about 13 months from diagnosis. Stable tumor growth for the next 63 months. Gradual increase in cyst size throughout the period. |
|  |  |  |  |  | CNS2 | **Spinal hb** (level of T9) | 13 months (A) | No | **Steady growth** over 13 months. |

Hb = Hemangioblastoma

***^1^** Stuttering growth pattern: defined as tumor growth characterized by periods of growth and periods of arrested growth ^1,2^.

***^2^** Acute debut of symptoms led to imaging at which 3 CNS hemangioblastomas were diagnosed (CNS1, CNS2, CNS3), unknown which tumor caused the specific symptoms.

In the following section are shown the sequenced *VHL* region for each sample with a somatic second hit variant detected, as seen in the bam files. The figures show the *VHL* variants detected and the sequencing reads in the area around the changed nucleotide. The reads are in green or blue color depending on the left or right orientation. The “read depth” in grey in the upper panel indicates the number of unique reads (sequences coming from different molecules and not PCR duplicates). The “net” over the sequence in the upper panel is the amount of reads originating from PCR duplicates, and they were not taken into account. The change in color over one nucleotide in the reads represents the variant allele (somatic mutation) detected by the software. Deletions are indicated by the pink color, while insertions by an orange column.

**Figure S1.** Binary alignment map showing reads over the somatic variant detected in patient 02, CNS2, *VHL*: c.488T>G; p.Leu163Arg.


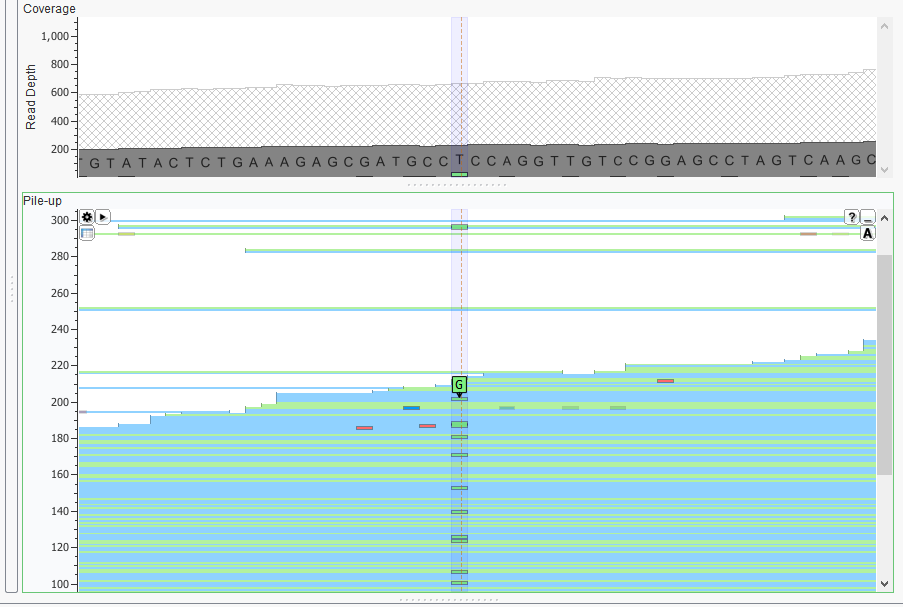


**Figure S2.** Binary alignment map showing reads over the somatic variant detected in patient 04, CNS2, *VHL*: c.234T>G; p.Asn78Lys


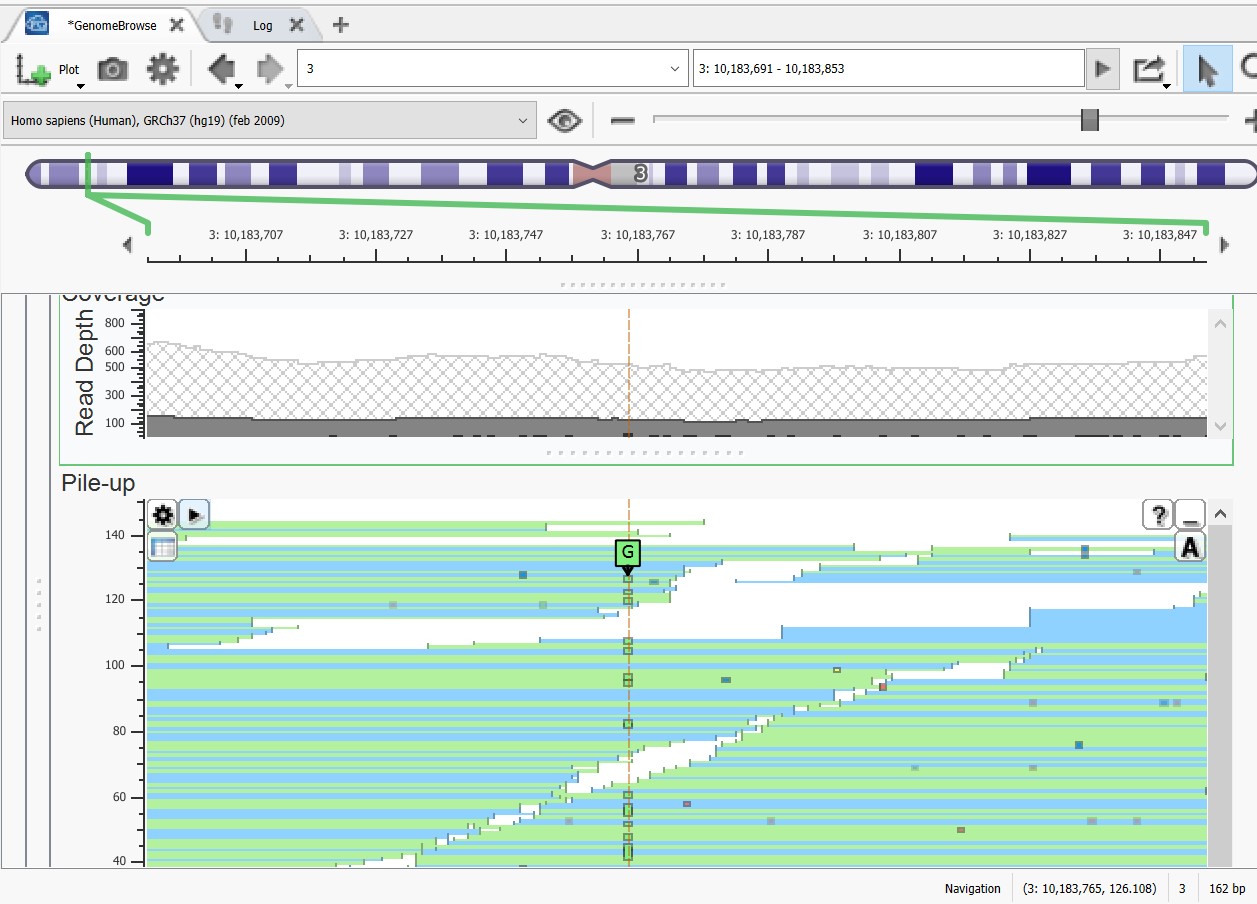


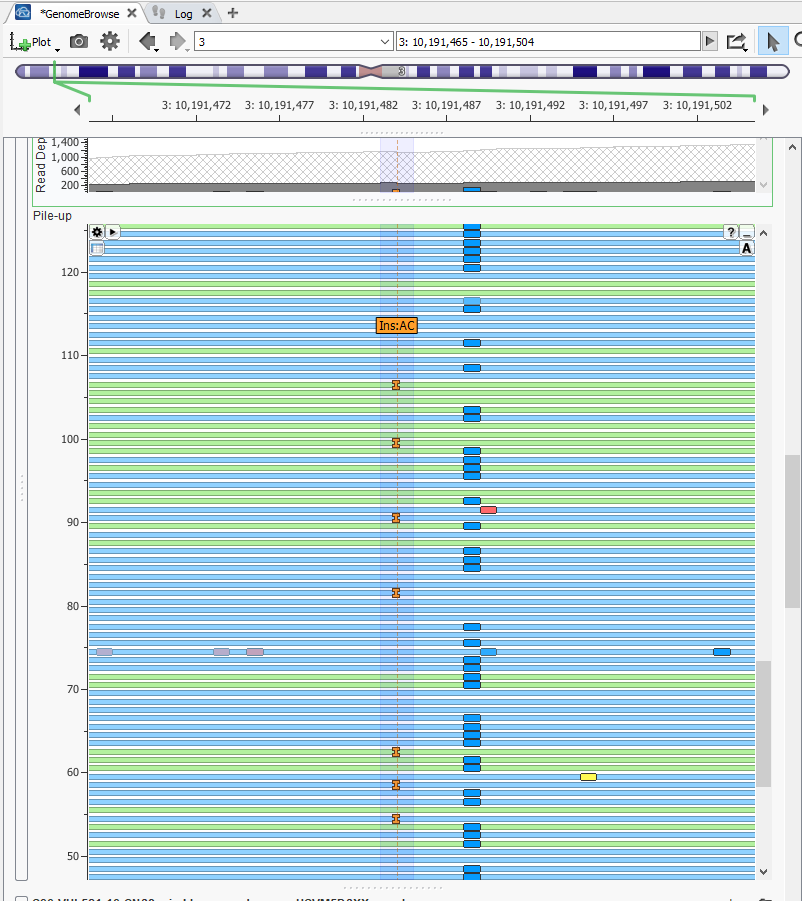
**Figure S3.** Binary alignment map showing reads over the variants detected in patient 05, CNS2, somatic *VHL:* c.477_478insCA; p.Glu160Glnfs*11 and germline *VHL*: c.481C>T; p.Arg161Ter.


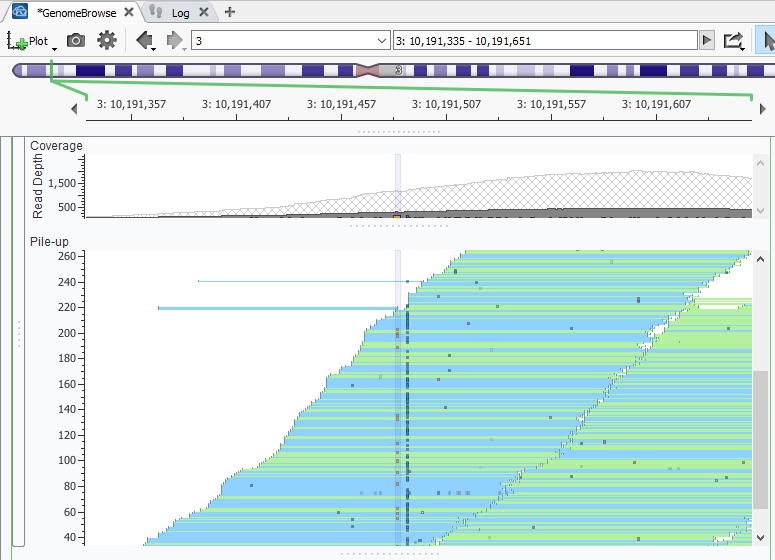
**Figure S4.** Binary alignment map showing reads over the *VHL* variants detected in patient 05, CNS2, larger view if the region.


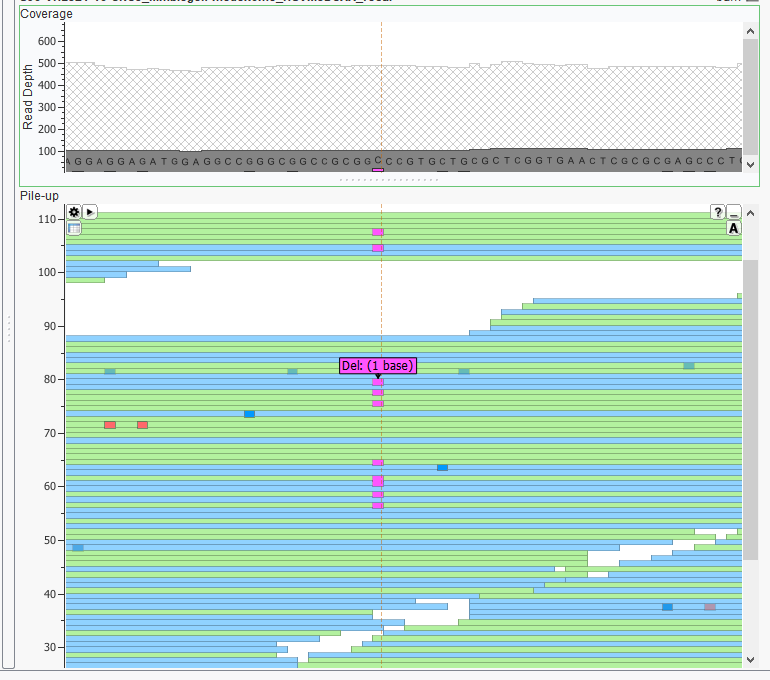
 **Figure S5.** Binary alignment map showing reads over the somatic variant in patient 05, CNS3, *VHL*: c.181delC; Val62Cysfs*5.

.

**Figure S6**. Binary alignment map showing reads over the somatic variant in patient 06, CNS1, *VHL*: c.454dupA; p.Thr152Asnfs*22 somatic insertion.


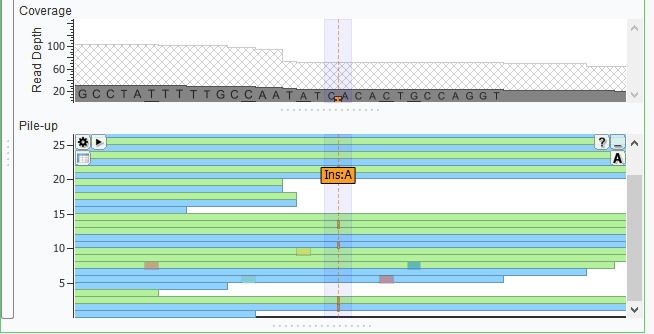


**Figure S7.** Binary alignment map showing reads over the somatic variant in patient 08, CNS1, *VHL*: c.634_635insGATGGAA; p.Gly212Glufs*46.


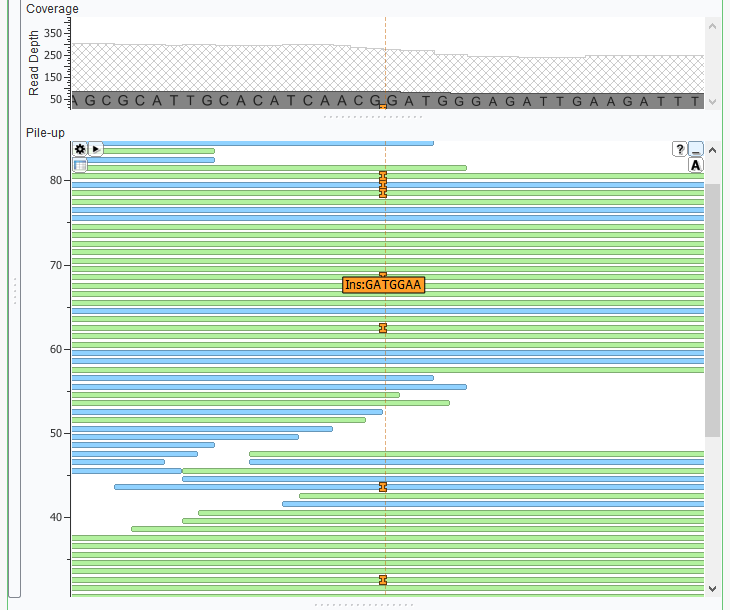


**Figure S8**. Binary alignment map showing reads over the somatic variant in patient 08, CNS1, larger view of the region.


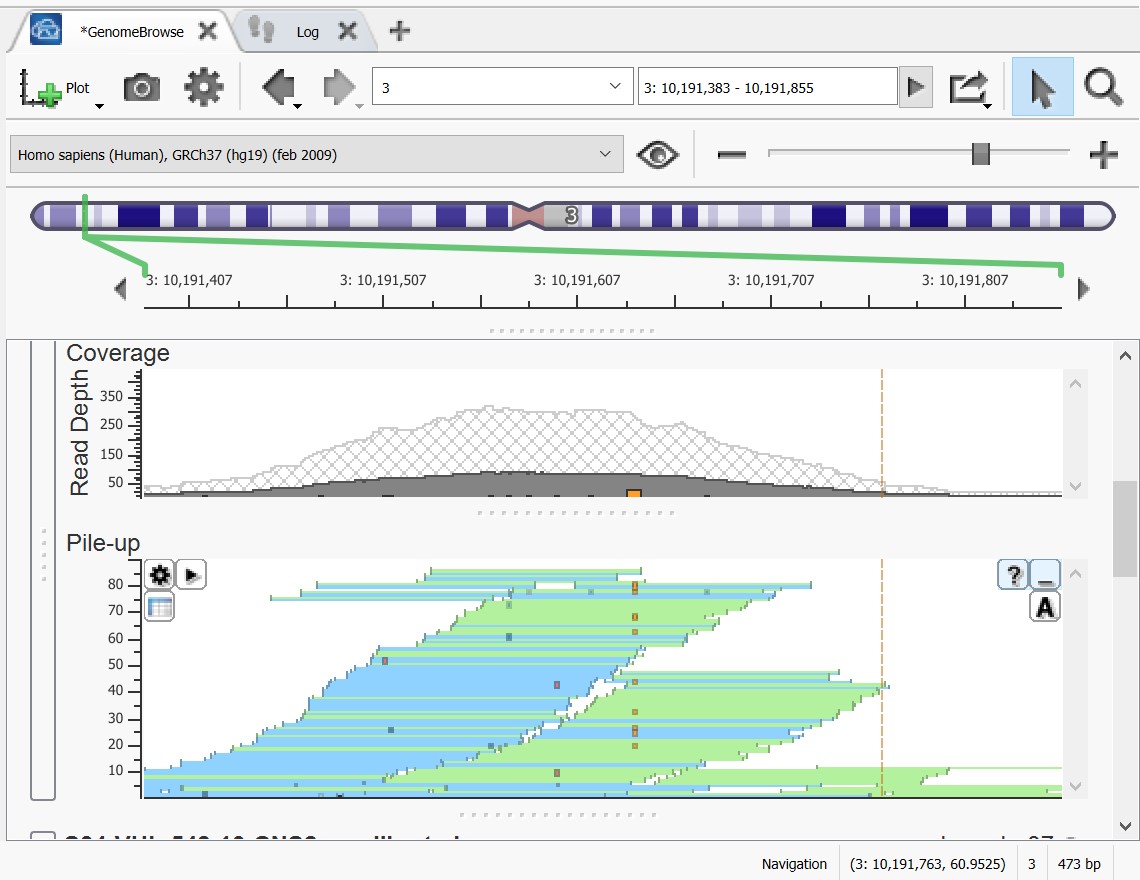


**Figure S9**. Binary alignment map showing reads over the somatic variant in patient 08, CNS2, *VHL*: c.462delA; p.Val155Cysfs*4 somatic deletion


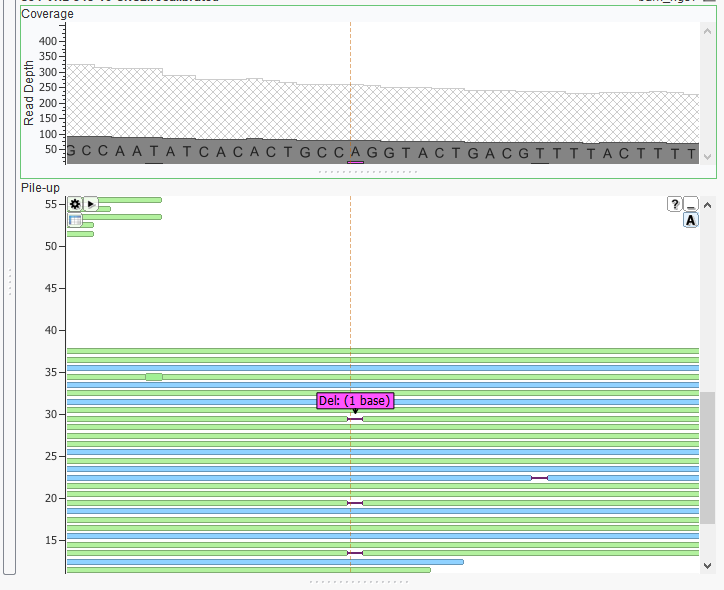


**Figure S10.** Binary alignment map showing reads over the somatic variant in patient 08, CNS2, larger view of the region.


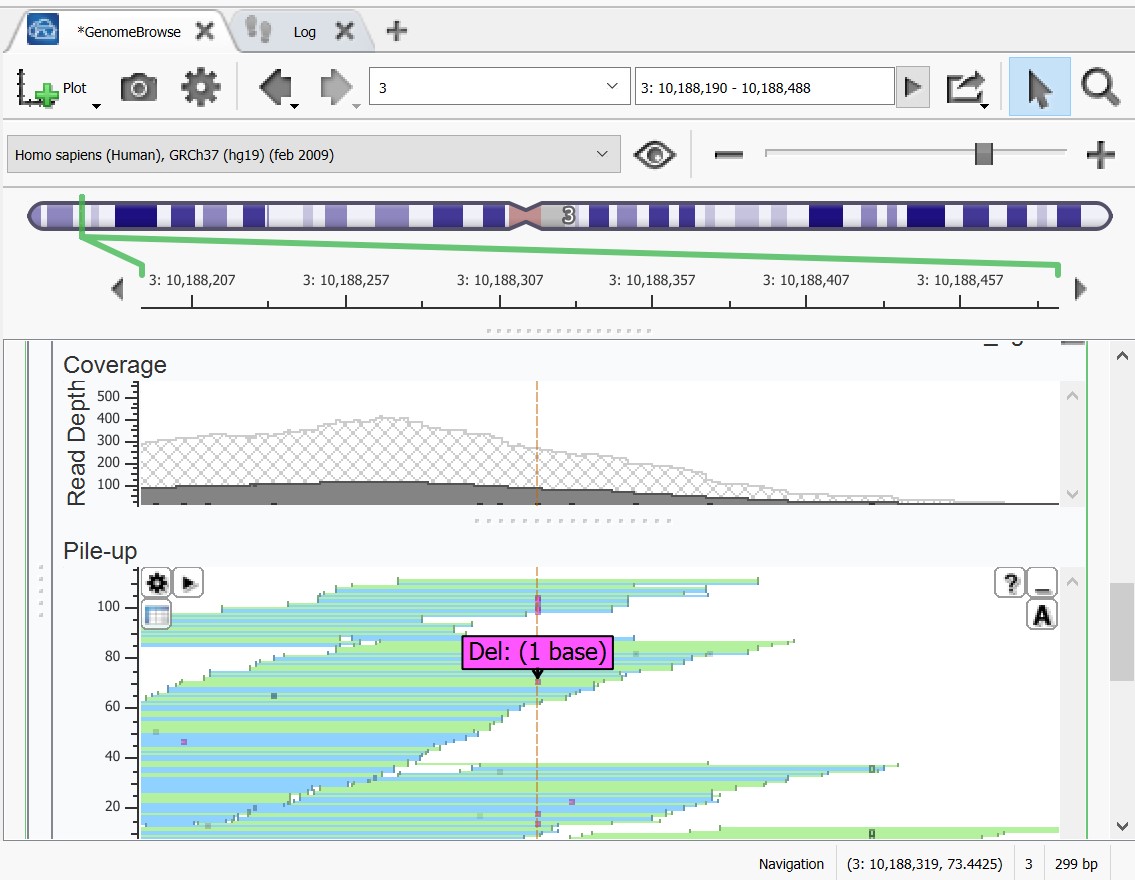


**Table S4.** Number of variants as identified by different variant callers

In the table are shown the number of variants from the vcf files according to CaVEMan, MuTect2, and Pindel variant caller algorithms. The number of variants after filtering, refers to the number of coding sequence and splicing site variants identified per sample after applying all quality and VarSeq filters. These include a low SOBDetector score of under 0.8, a “pass” designation in variant caller built-in filters, at least 25 reads over the region, a variant allele frequency (VAF) in reads higher than 0.1, and a manual check. The overlap between the two software refers to the variants identified by both algorithms.

|  |  |  | **Number of somatic variants in vcf files** | | | **Number of somatic variants after filtering** | | | **Overlap between CaVEMan and Mutect2** |
| --- | --- | --- | --- | --- | --- | --- | --- | --- | --- |
| **Patient** |  | **Sample** | **MuTect2** | **CaVEMan** | **Pindel** | **MuTect2** | **CaVEMan** | **Pindel** |  |
| 1 |  | CNS1 | 1330 | 1173 | 113 | 11 | 10 | 0 | 0 |
|  |  | CNS3 | 1720 | 7893 | 303 | 0 | 0 | 0 | 0 |
| 2 |  | CNS1 | 2624 | 1247 | 86 | 14 | 2 | 0 | 0 |
|  |  | CNS2 | 1816 | 2271 | 232 | 8 | 2 | 0 | 2 |
|  |  | CNS3 | 1600 | 8373 | 198 | 6 | 5 | 0 | 4 |
| 3 |  | CNS1 | 2379 | 6317 | 218 | 10 | 7 | 0 | 5 |
| 4 |  | CNS1 | 2118 | 7926 | 149 | 6 | 5 | 0 | 4 |
|  |  | CNS2 | 2808 | 5136 | 236 | 23 | 16 | 0 | 14 |
|  |  | CNS3 | 1333 | 676 | 27 | 0 | 2 | 0 | 0 |
| 5 |  | CNS1 | 2829 | 538 | 26 | 1 | 0 | 0 | 0 |
|  |  | CNS2 | 2843 | 1740 | 218 | 1 | 0 | 0 | 0 |
|  |  | CNS3 | 2508 | 2305 | 199 | 2 | 0 | 0 | 0 |
|  |  | CNS4 | 2568 | NA | 147 | 1 | 0 | 0 | 0 |
|  |  | CNS5 | 2866 | 4251 | 178 | 2 | 2 | 0 | 0 |
| 6 |  | CNS1 | 1854 | 1532 | 56 | 15 | 3 | 0 | 0 |
|  |  | CNS3 | 2469 | 2804 | 126 | 10 | 4 | 0 | 1 |
|  |  | CNS4 | 1495 | 2060 | 72 | 5 | 1 | 0 | 0 |
|  |  | CNS6 | 2115 | 3798 | 193 | 1 | 0 | 0 | 0 |
|  |  | CNS7 | 1796 | 4836 | 178 | 0 | 0 | 0 | 0 |
|  |  | CNS9 | 1519 | 4905 | 123 | 11 | 6 | 0 | 5 |
| 8 |  | CNS1 | 1358 | 1453 | 2849 | 1 | 1 | 0 | 0 |
|  |  | CNS2 | 1274 | 8457 | 544 | 1 | 6 | 0 | 1 |
|  |  | **Average** | **2055,55** | **3794,81** | **294,14** | **6** | **3** | **0** | **2** |
|  |  | **Total** |  |  |  | **129** | **72** | **0** | **36** |

**Figure S11.** Whole genome profiles of all 22 samples: results from the ASCAT analysis


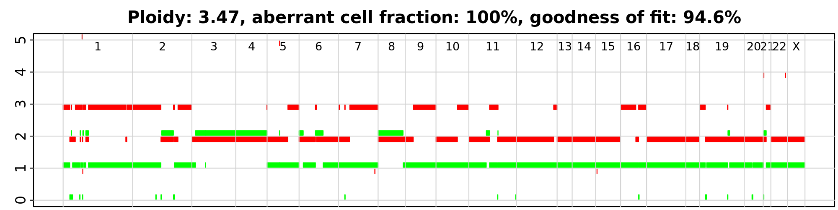
Sample 01 CNS1

02 CNS1

01 CNS3

01 CNS1


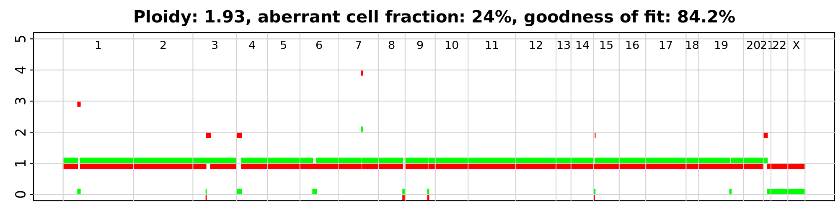
Sample 01 CNS3

Sample 02 CNS1
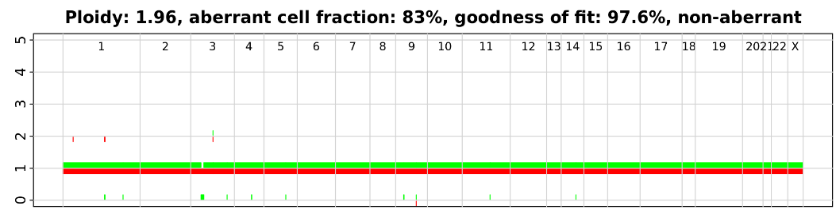


Sample 02 CNS2


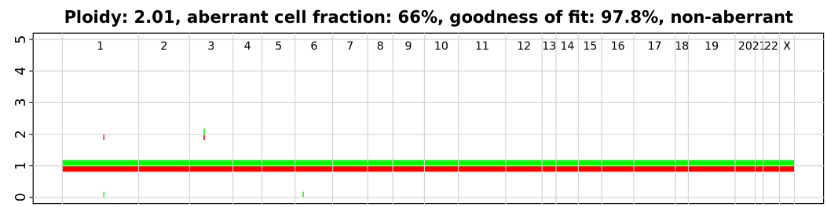


Sample 02 CNS3


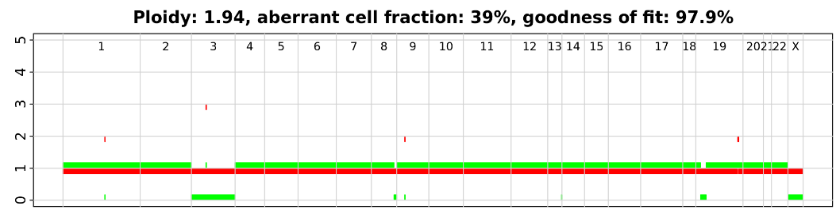


Sample 03 CNS1


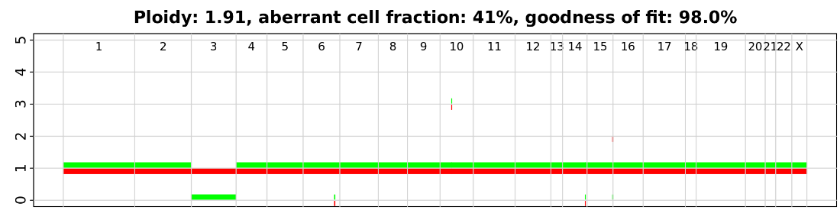


Sample 04 CNS1


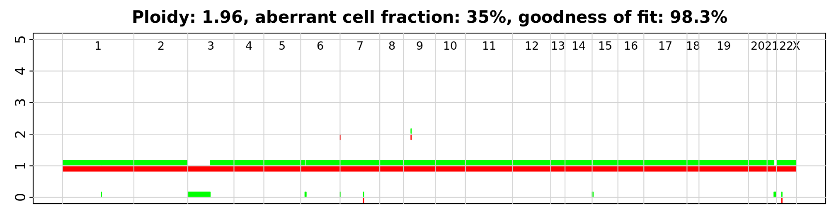


Sample 04 CNS2


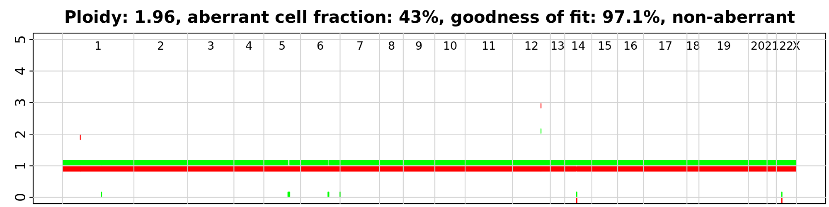


Sample 04 CNS3 – no solution

Sample 05 CNS1 – no solution

Sample 05 CNS2


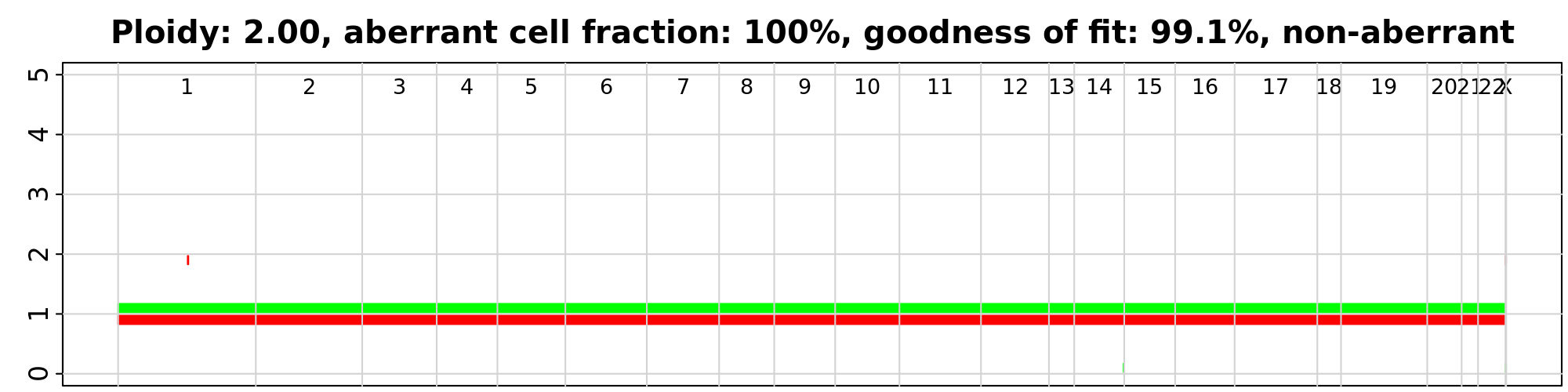


Sample 05 CNS3


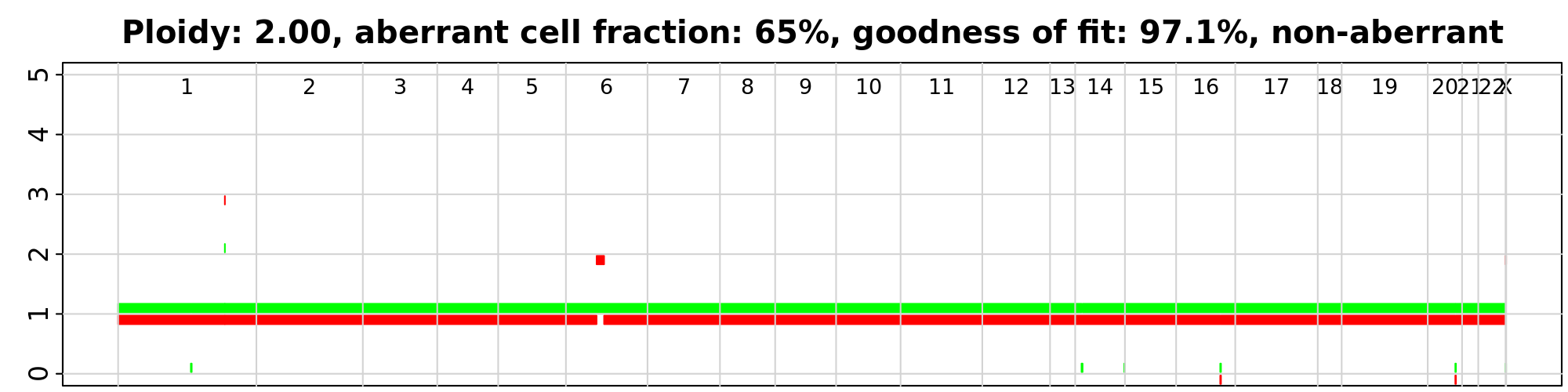


Sample 05 CNS4


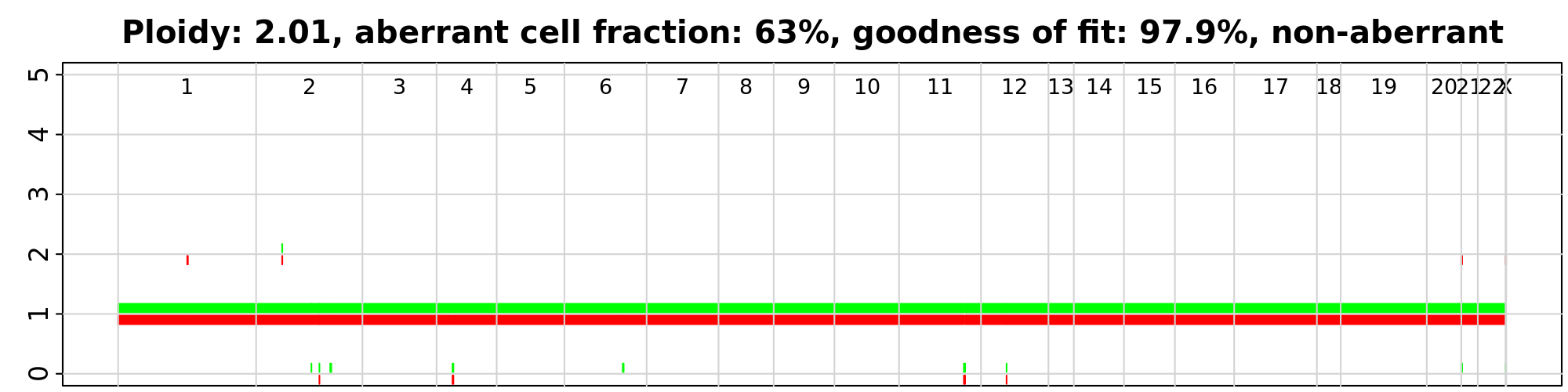


Sample 05 CNS5


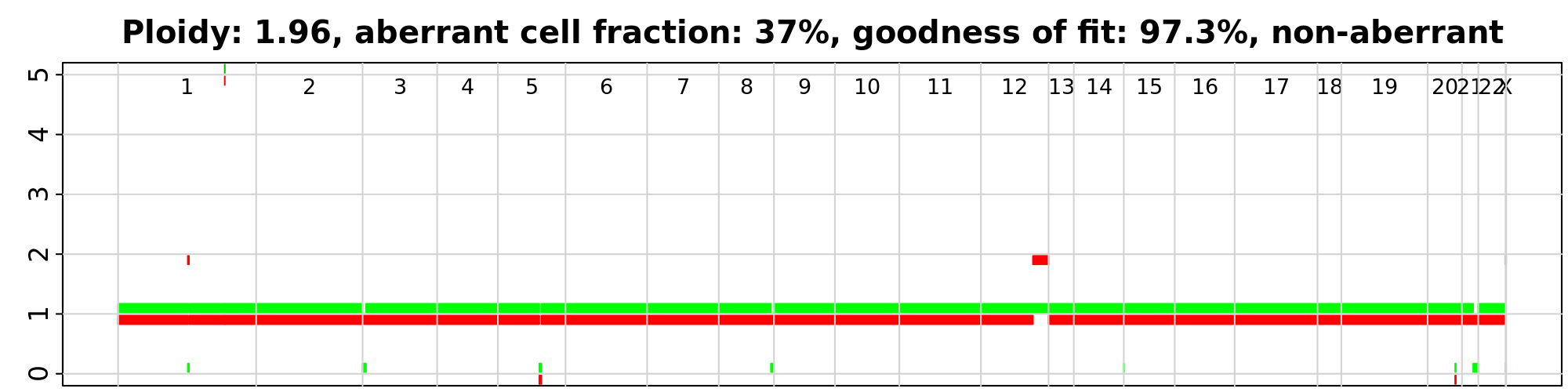


Sample 06 CNS1


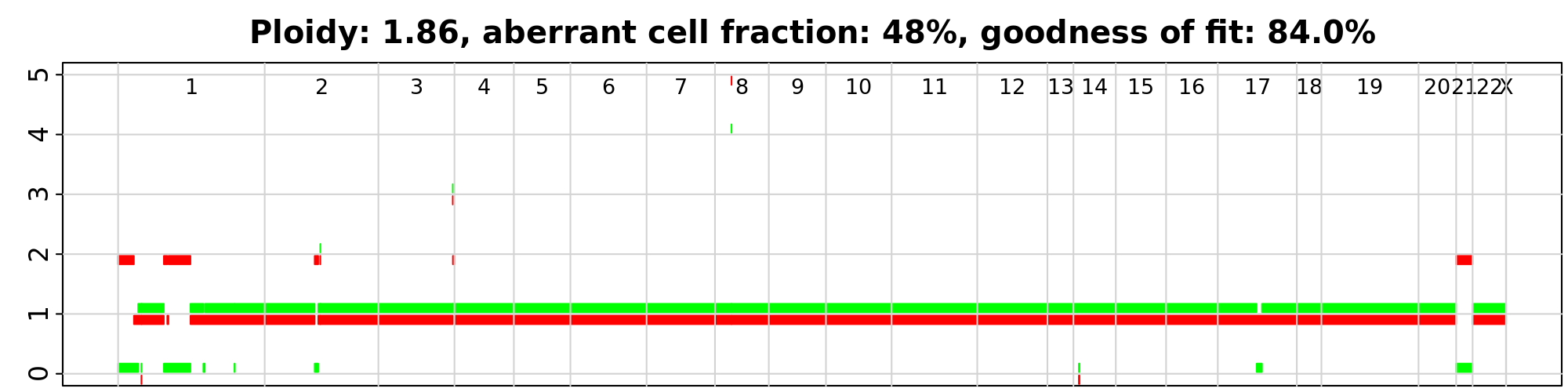


Sample 06 CNS3


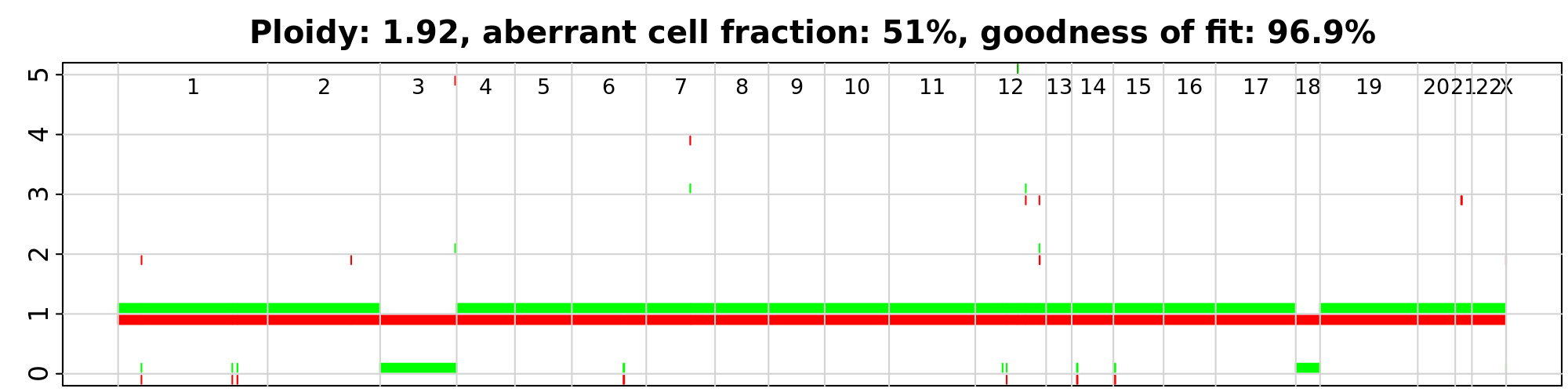


Sample 06 CNS4


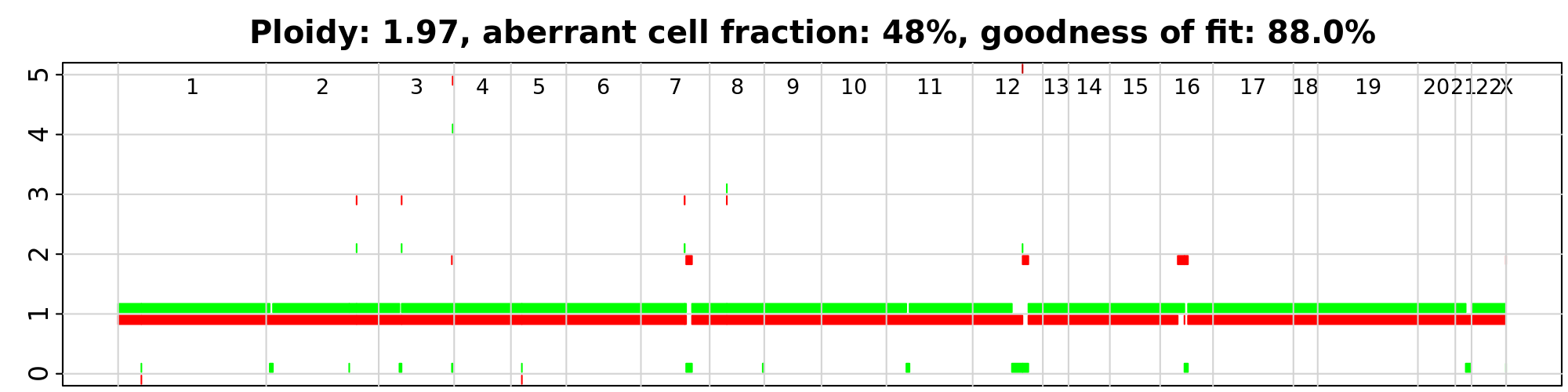


Sample 06 CNS6


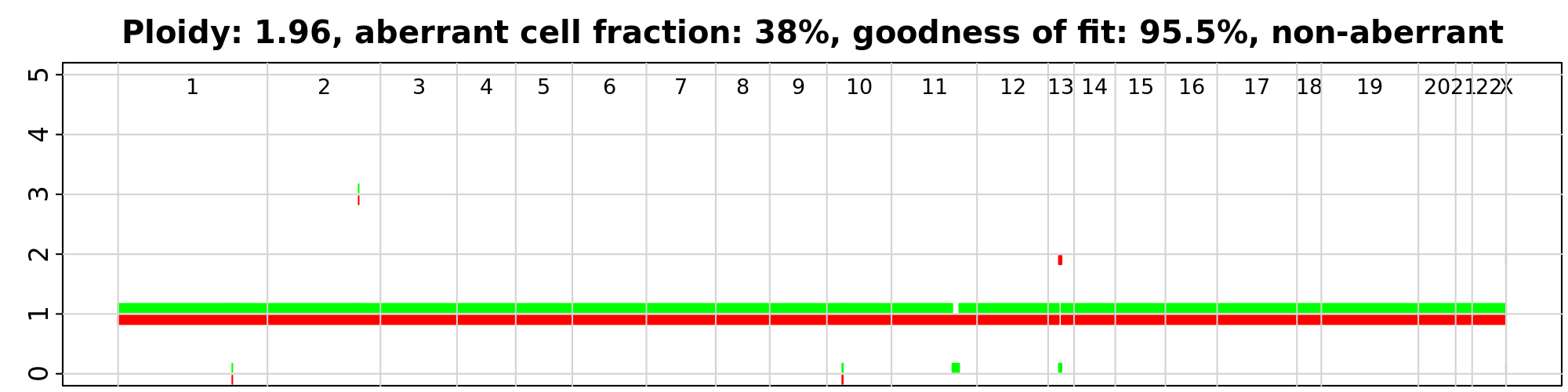


Sample 06 CNS7


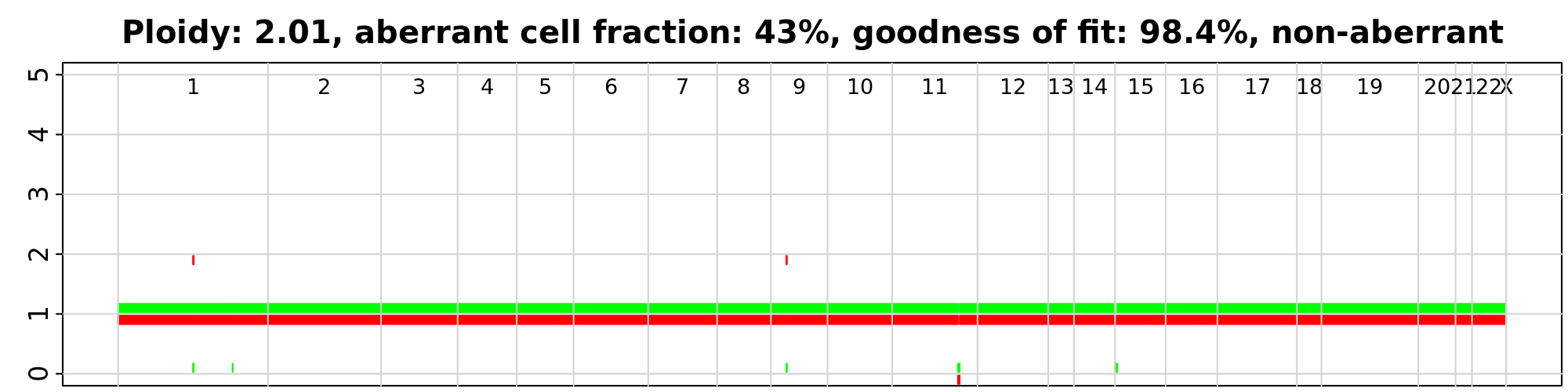


Sample 06 CNS9
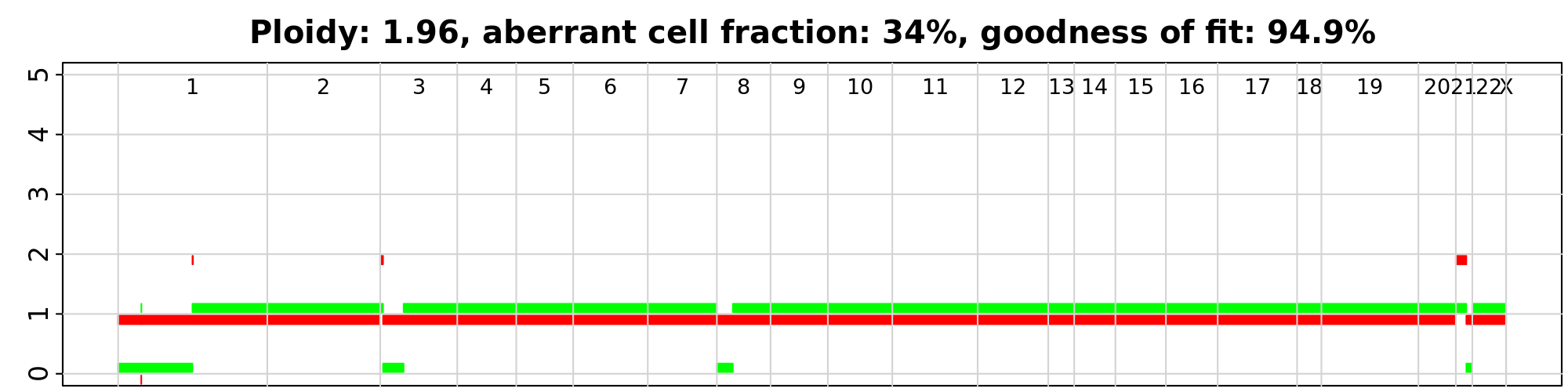


Sample 08 CNS1


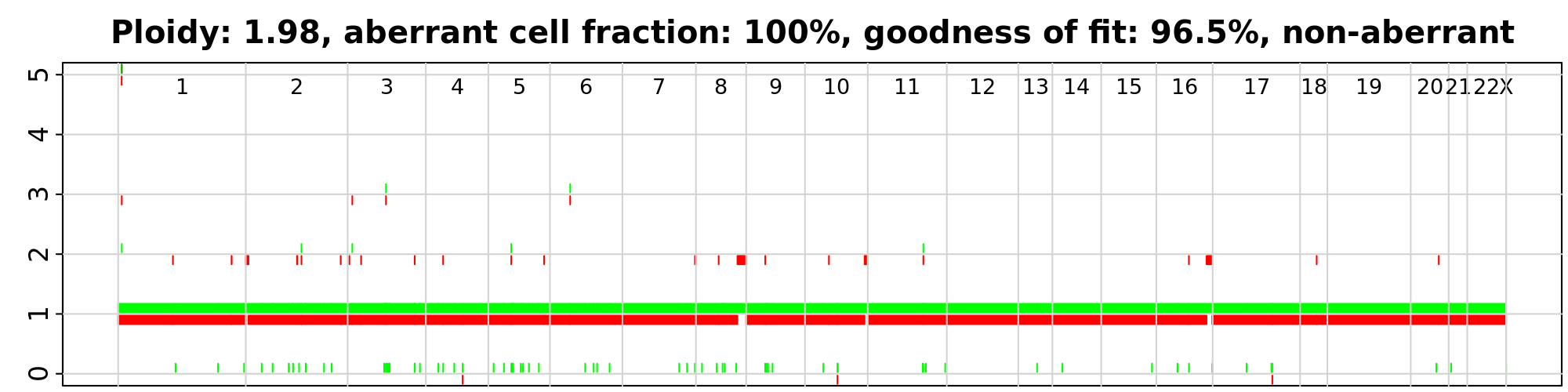


Sample 08 CNS2


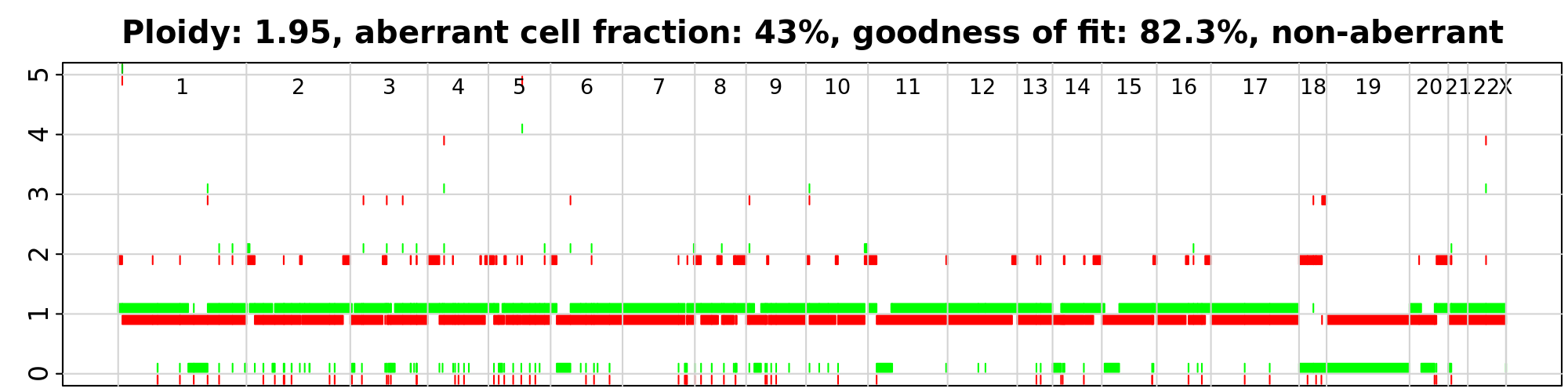

Supplement: Supplementary file 1 — Fig. S1. Binary alignment map showing reads over the somatic variant detected in patient 02, CNS2, VHL: c.488T>G; p.Leu163Arg. Fig. S2. Binary alignment map showing reads over the somatic variant detected in patient 04, CNS2, VHL: c.234T>G; p.Asn78Lys. Fig. S3. Binary alignment map showing reads over the variants detected in patient 05, CNS2, somatic VHL: c.477_478insCA; p.Glu160Glnfs*11 and germline VHL: c.481C>T; p.Arg161Ter. Fig. S4. Binary alignment map showing reads over the VHL variants detected in patient 05, CNS2, larger view if the region. Fig. S5. Binary alignment map showing reads over the somatic variant in patient 05, CNS3, VHL: c.181delC; Val62Cysfs*5. Fig. S6. Binary alignment map showing reads over the somatic variant in patient 06, CNS1, VHL: c.454dupA; p.Thr152Asnfs*22 somatic insertion. Fig. S7. Binary alignment map showing reads over the somatic variant in patient 08, CNS1, VHL: c.634_635insGATGGAA; p.Gly212Glufs*46. Fig. S8. Binary alignment map showing reads over the somatic variant in patient 08, CNS1, larger view of the region. Fig. S9. Binary alignment map showing reads over the somatic variant in patient 08, CNS2, VHL: c.462delA; p.Val155Cysfs*4 somatic deletion. Fig. S10. Binary alignment map showing reads over the somatic variant in patient 08, CNS2, larger view of the region. Fig. S11. Whole genome profiles of all 22 samples: results from the ASCAT analysis. Table S1. Clinical characteristics of included vHL patients and CNS hemangioblastomas. [file MOL2-9999-0-s004.docx]
